# Mental health during the COVID-19 pandemic and first lockdown in Lebanon: Risk factors and daily life difficulties in a multiple-crises setting

**DOI:** 10.1371/journal.pone.0297670

**Published:** 2024-02-16

**Authors:** Martine Elbejjani, Sara Mansour, Rawan A. Hammoud, Catrina Ziade, Batoul Assi, Ahmad Assi, Samya El Sayed, Rita El Hachem, Hala Kerbage

**Affiliations:** 1 Clinical Research Institute, Department of Internal Medicine, Faculty of Medicine, American University of Beirut, Beirut, Lebanon; 2 Faculty of Medicine, American University of Beirut, Beirut, Lebanon; 3 Saint-Eloi University Hospital, Montpellier, France; 4 INSERM U 1018 Developmental Psychiatry, Paris-Saclay University, Gif-sur-Yvette, France; University of Bern: Universitat Bern, SWITZERLAND

## Abstract

**Introduction:**

Research from around the world shows important differences in the impact of the COVID-19 pandemic and lockdowns on mental health. This study examined the extent of mental health challenges (depressive and anxiety symptoms and daily life difficulties) and their associations with pandemic- and response-related factors during the first lockdown in Lebanon, which happened amid a severe economic crisis and socio-political turmoil.

**Methods:**

Data come from a cross-sectional internet-based survey (May-June 2020). Association of depressive (Patient Health Questionnaire (PHQ-9)) and anxiety symptoms (Generalized Anxiety Disorder (GAD-7)) with outbreak-related worries and knowledge, information sources, and confidence and satisfaction in response measures were estimated using logistic regression adjusted for sociodemographic and socioeconomic indicators.

**Results:**

Among 510 participants (mean age 36.1±11.3; 69.4% women), 32.3% had elevated depressive and 27.3% had elevated anxiety symptoms; younger age, unemployment, loss of employment, and lower income were related to more mental health symptoms. Most prevalent daily life challenges were feelings of uncertainty (74.5%) and financial (52.2%) and emotional (42.2%) difficulties; these and all other daily life difficulties (work-related, caregiving, and online learning) were significantly higher among participants with higher depressive and anxiety symptoms. Higher outbreak-related worries were associated with higher depressive (OR = 1.36, 95% CI = 1.20,1.53) and anxiety symptoms (OR = 1.47, 95% CI = 1.30,1.67). Higher pandemic-related knowledge, reliance on and trust in local health agencies and professionals’ information, and satisfaction and confidence regarding governmental and health institutions’ response were all related to lower mental health symptoms.

**Conclusion:**

Results show that mental health burden in Lebanon during the first lockdown (when the COVID-19 outbreak was still minimal) is among the highest reported worldwide and highlight elevated emotional and financial tolls and widespread impact on daily life. In this high-burden and multiple-challenges context, results suggest an important role for the healthcare body, knowledge, and trust in the institutions managing the response.

## Introduction

Three years on and the coronavirus disease 2019 (COVID-19) pandemic continues to be a global health problem. In addition to its large toll on mortality and physical illness [1], the pandemic’s impact on mental health has been substantial [2]. The pandemic and related response measures, notably lockdowns, triggered significant disruptions in daily life and had far-reaching consequences, particularly on people’s well-being worldwide [3, 4]. Studies conducted early in the pandemic found a higher prevalence of depressive and anxiety symptoms compared to pre-pandemic trends, with varying estimates across populations [2], ranging from 14.7% to 42% for anxiety and 18.7% to 39% for depression [5–9]. This uneven impact on mental health is thought to be explained by the heterogeneity in the severity of the pandemic and stringency of response measures, in addition to differences in individual and contextual factors such as socio-economic conditions, existing social inequalities, and cultural attitudes.

Current evidence puts forward a wide spectrum of factors that influenced mental health during the COVID-19 pandemic and lockdowns. One German study identified fear of getting infected and consequences for oneself or loved ones as significant stressors associated with mental health problems in the earlier phase of the pandemic [10]. Data from the March 2020 lockdown in Spain showed that experiencing infection symptoms, having an infected close relative, and younger age were associated with higher anxiety and depressive symptoms [11]. Another study from Ireland reported that female gender and loss of income due to the pandemic were related to worse mental health symptoms [12]. Lack of knowledge and uncertainty about the pandemic were risk factors for poorer mental health among American samples [13]. Confidence in doctors, satisfaction with health information, and knowledge of preventive measures were found to be protective for mental health among the general population in China [14]. Whereas, getting outside more often, the practice of religiosity, and perceived social support were found to be protective in representative samples of U.S. adults [15].

In the context of such variability and with the continual threat of the emergence of new variants with higher spread and immunity evasion potential, there is a great need for investigations that systematically assess multifaceted sources of distress. Such efforts are instrumental for identifying priority modifiable risk factors and venues for both pandemic and mental health mitigation interventions. Further, given that both the pandemic and response measures heightened several key risk factors for mental health problems (e.g., worsening socioeconomic conditions and destabilizing daily life and resources) [16], it is important to go beyond estimating the prevalence of anxiety and depressive symptoms to characterize the specific difficulties and hardships collectively experienced. These data are particularly needed in low-resource and multiple-crisis settings, where the pandemic imposed further strains on limited economic and healthcare resources and higher increase in depression and anxiety prevalence [17].

The COVID-19 outbreak began in Lebanon in end of February 2020, when the country was already a few months into its worst economic crisis and alarmingly deteriorating living conditions [18]. To date, a few studies examined mental health challenges during the pandemic in Lebanon, reporting elevated prevalence of depression and anxiety and a role for fear of the outbreak and economic insecurity in worsening mental health symptoms [19–21]. In this study, we examined the extent and correlates of depressive and anxiety symptoms during the first country-wide lockdown in Lebanon, with a specific focus on characterizing daily life difficulties and challenges that Lebanese residents faced and on systematically investigating how several components of the pandemic and response measures were related to mental health symptoms. Specifically, we examined both general and specific daily life difficulties, social support and protective factors, and the role of COVID-19-related worries, knowledge, sources of information, and satisfaction and confidence regarding government and health agencies’ response.

## Materials and methods

### Study design and participants

The study consists of a cross-sectional internet-based survey conducted between May 2 and June 8, 2020, following the 3-month first nationwide lockdown in Lebanon, which was set on March 10, 2020, with lockdown measures gradually easing off in the first week of May 2020. Eligibility criteria were: i) being 18 years or older and ii) residing in Lebanon for the duration of this first government-mandated lockdown.

The study recruited participants by disseminating the survey in both Arabic and English (LimeSurvey) on several online communication and social media platforms, including WhatsApp, Facebook, Twitter, and Instagram. Recruitment was based on convenience sampling with an invitation to complete and/or share the survey, allowing for snowball sampling. The study was approved by the Institutional Review Board of the American University of Beirut (SBS-2020-0208), and participants provided a consent to participate (digital consent) prior to accessing the survey.

### Sample size calculation

We estimated a sample size of 320 to 350 to detect differences in the effect size of 0.4 and OR of 1.5 between those with and without elevated mental health symptoms (similar to previously reported associations of depression and anxiety risk with social and socioeconomic indicators) [22–25], with an alpha of 0.05 and 85% power and assuming prevalence of elevated mental health symptoms of 20 to 25%. Our recruitment strategy aimed to maximize the number of participants, so we allowed recruitment beyond the estimated target sample size.

### Measures

The survey collected data on mental health outcomes, demographic and socioeconomic factors, difficulties experienced and sources of support during the first lockdown, and on pandemic and response related factors, as detailed below.

#### Depressive and anxiety symptoms

Depressive and anxiety symptoms were measured using the Patient Health Questionnaire-9 (PHQ-9) and the Generalized Anxiety Disorder -7 (GAD-7) scales, two widely used instruments that measure the presence and severity of depressive and anxiety symptoms, respectively [26, 27]. Both scales’ items are based on the DSM-IV criteria and are rated in frequency of occurrence over the past two weeks (on a 4-point Likert scale). The total PHQ-9 score is the sum of all 9 item scores (ranging from 0 to 27), with higher scores indicating more depressive symptoms and scores ≥10 indicating the presence of elevated depressive symptoms [28]. The total GAD-7 score is a sum of the 7 item scores, ranging from 0 to 21, with higher scores indicating higher anxiety symptoms and scores ≥10 indicating elevated anxiety symptoms [28]. PHQ-9 has high internal consistency (Cronbach’s alpha between .86 and .88) [29] and high test reliability (Cronbach’s alpha between .84 and .95) [26, 30]; GAD-7 exhibits excellent internal consistency (Cronbach’s alpha between .89 and .92) [27, 30, 31].

Both scales have been adapted and validated in Lebanon and Arab-speaking communities [26], and the validated versions were used in this survey.

#### General and specific daily life difficulties

Participants reported the extent to which they faced general and specific daily life functioning difficulties since the first lockdown (on a 5-point Likert scale from (1) not at all to (5) extremely). General difficulties included 10 items: emotional difficulties (feeling sad, stressed, demotivated), fear due to circumstances, uncertainity regarding the future, social isolation (unable to see family and friends), boredom, financial insecurity, food insecurity (unable to get basic food, water, and hygiene items), medical worries (unable to get regular medical care and prescription), physical activity (unable to walk or do exercises) and diet worries (experiencing diet changes); detailed items presented in S2 Table. Specific difficulties assessed presence and extent of difficulties with: work (work totally suspended because cannot be there in person, not having adequate resources to work from home, not being able to focus, worrying about losing job), caregiving responsibilities (for children and/or older relatives), and learning-related responsibilities (learning online, having to help children with online learning). A composite score of general difficulties was the count of items rated as very much or extremely difficult for each participant, generating a total general difficulties score (ranging from 0 to 10). A composite frequency score for the specific difficulties (ranging from 0 to 100%) was generated, indicating the frequency of greater specific difficulties, by first summing the number of items rated as very much/extremely difficult and then dividing them by the total number of specific difficulties reported (i.e., whether extreme or not), given that specific daily life difficulties were not applicable to all participants (e.g., learning, caregiving, work responsibilities).

#### Support sources

Participants also rated the importance of the following support sources since the beginning of lockdown (from not at all (1) to very important (5)): Family support, friend support, personal hobbies, house chores (cooking, cleaning, tidying, etc.), exercising or playing sports, religious activities and praying, spirituality (meditation, yoga, doing relaxation activities, etc.), work or school, having an outdoor space at home (balcony, garden, etc.), and social media platforms. A composite score of support sources (ranging 0–10) was computed by summing the number of support sources rated as important/very important.

#### Pandemic- and response-related factors

*Worries about the COVID-19 infection*. The survey assessed the extent (from 1: not at all to 5: extremely) participants worried about: spreading the virus to others, contracting COVID-19 infection themselves, health complications from the infection, isolation, not being able to receive proper treatment and care, financial consequences, and stigma if they were to contract the virus. A composite score of COVID-19-related worries was the count of items rated as very much/extremely worrisome, generating a 0 to 7 total score, with higher scores indicating more worries.

*COVID-19 related knowledge*. Participants rated their knowledge about the COVID-19 infection (i) prevention, (ii) symptoms, and (iii) spread (each rated from (1) not at all to (5) very knowledgeable). Each item was then dichotomized into high (answers of knowledgeable/very knowledgeable) vs low knowledge.

*Sources of information and related trust*. Data on the main sources of information about the COVID-19 pandemic were collected (refer to Table 1). For each information source, participants ranked the frequency (from 1: never to 5: all the time) they received COVID-19 information from that source and the extent they trusted the information from this source (ranging from 1: not at all to 5: extremely).

**Table 1:** Distribution of the reported difficulties and other outbreak/lockdown-related factors in the total sample.

Sample characteristics	Mean (SD) or n (%)	Missing, n (%)
**Age,** in years	36.11 (11.26)	
**Gender**		
Male	146 (28.63)	
Female	354 (69.41)	
Other/prefer not to answer	10 (1.96)	
**Marital status**		
Single/widow/divorced	281 (55.10)	
Engaged/married	225 (44.12)	
Prefer not to answer	4 (0.78)	
**University Degree**		
Yes	468 (91.76)	
No	37 (7.25)	
Prefer not to answer	5 (0.98)	
**Employment status**		
Employed (Full-time/part-time/self- employed/retired/homemaker/student)	485 (95.10)	
Unemployed (unemployed and seeking work, unemployed and not seeking work)	25 (4.90)	
**Employment change**		
Loss of job		
Yes	15 (2.94)	
No	495 (97.06)	
**Change in income since lockdown**		
No income before/after	92 (18.04)	
Stopped/decreased	217 (42.55)	
Stayed the same/increase	201 (39.41)	
**Mental health and general/specific daily life difficulties**	**High extent**^**c**^ n (%^**a**^)	**Missing,** n (%)
**Mental health symptoms**		
Depression, elevated symptoms (PHQ-9 score ≥ 10)	165 (32.3)	
Anxiety, elevated symptoms (GAD-7 score ≥10)	139 (27.3)	
**General difficulties**		
Uncertainty regarding the future	380 (74.51)	
Financial insecurity	266 (52.16)	
Emotional	215 (42.16)	
Fear from circumstances	197 (38.63)	
Social Isolation	189 (37.06)	
Boredom	133 (26.08)	
Physical activity	128 (25.10)	
Diet worries (diet changes)	121 (23.73)	
Medical worries	82 (16.08)	
Food insecurity	75 (14.71)	
**Specific daily life difficulties**		*Not applicable*^***b***^, *n (%)*
Work-related:		
*Not being able to focus*	130 (32.34)	108 (21.18)
*Worrying about losing my job*	107 (27.65)	123 (24.12)
*Work suspended because cannot be there in person*	97 (25.94)	136 (26.67)
*Not having adequate resources to do work from home*	66 (17.60)	135 (26.47)
Caregiving responsibilities	120 (31.41)	128 (25.10)
Having to help children with online learning	77 (25.41)	207 (40.59)
Learning online	79 (22.77)	163 (31.96)
**Outbreak/lockdown-related factors**	**High extent**^**c**^ n (%^**a**^)	**Missing** ^**d**^, n (%)
**Sources of support**		
Family support	374 (73.33)	
Having an outdoor space at home (balcony, garden, rooftop…)	349 (68.43)	
Friend support	306 (60.00)	
House chores (cooking, cleaning, tidying,. . .)	270 (52.94)	
Personal hobbies	268 (52.55)	
Work or School	240 (47.06)	
Exercising or playing sports	228 (44.71)	
Being on social media platforms	212 (41.57)	
Religious activities/praying	167 (32.75)	
Relaxation activities (Meditation, yoga…)	115 (22.55)	
**Outbreak-related worries**		
Spreading the virus to others	409 (80.20)	
Not getting proper care/treatment	179 (35.10)	
Isolation	167 (32.75)	
Contracting the virus myself	150 (29.41)	
Health complications from infection	149 (29.22)	
Serious financial consequences of infection	129 (25.29)	
People’s negative reaction	111 (21.76)	
**Knowledge about the pandemic**		
Knowledge about prevention	416 (84.04)	15 (2.94)
Knowledge about symptoms	390 (78.79)	15 (2.94)
Knowledge about spread	309 (62.42)	15 (2.94)
Mostly seeing conflicting news	224 (45.25)	15 (2.94)
**Sources of information about COVID-19**		
Regional and international health agencies (WHO, CDC, Worldometer)	295 (59.59)	15 (2.94)
Lebanese Ministry of Public Health communications (website, social media, application)	269 (54.34)	15 (2.94)
People you speak to daily (family, friends, colleagues)	261 (52.72)	15 (2.94)
Local Media (TV stations, newspapers, news platforms and their websites, social media accounts, radio, and applications)	260 (52.53)	15 (2.94)
Public opinions on social media (WhatsApp, Facebook, Instagram, Twitter)	188 (37.98)	15 (2.94)
Healthcare professionals (doctor, pharmacist)	177 (35.76)	15 (2.94)
**Trust in the sources of information**		
Regional and international health agencies (WHO, CDC, etc.)	338 (68.42)	16 (3.14)
Healthcare professionals (doctor, pharmacist)	337 (68.36)	17 (3.33)
Lebanese Ministry of Public Health communications (website, social media, application)	240 (48.58)	16 (3.14)
Local Media (TV stations, newspapers, news platforms and their websites, social media accounts, radio, and applications)	135 (27.33)	16 (3.14)
People you speak to daily (family, friends, colleagues)	124 (25.10)	16 (3.14)
Public opinions on social media (WhatsApp, Facebook, Instagram, Twitter)	23 (4.66)	16 (3.14)
**Confidence with different sectors’ handling of the pandemic**		
Confidence in the Lebanese Ministry of Public Health	207 (43.13)	30 (5.88)
Confidence in the Lebanese Government	177 (37.03)	32 (6.27)
Confidence in the health services in Lebanon	173 (35.89)	28 (5.49)
**Satisfaction with different sectors’ handling of the pandemic**		
Satisfaction with the Lebanese Ministry of Public Health	251 (51.97)	27 (5.29)
Satisfaction with health services in Lebanon	247 (51.67)	32 (6.27)
Satisfaction with the Lebanese Government	230 (47.62)	27 (5.29)

SD: standard deviation

Means (standard deviations) are presented for continuous variables. Frequencies (percentages) are presented for categorical variables.

^**a**^ percentages estimated on total complete observations (excluding missing observations).

^**b**^ a whether specific daily life functioning item were not applicable to them (i.e., working, caregiving).

^**c**^ for depressive and anxiety symptoms, high extent indicates scores ≥10 on Patient Health Questionnaire-9 and the Generalized Anxiety Disorder-7 scales respectively; for all other items, high extent indicates ratings of the highest two levels (e.g., difficult/very difficult; knowledgeable/very knowledgeable; important/very important).

^**d**^ missing values for the satisfaction/confidence items include missing observations (≤16 observations (3.14%)) and those with an answer of no opinion (≤17 observations (3.33%)).

Participants also reported whether they have mostly seen conflicting information about COVID-19 (yes/no).

*Satisfaction and trust regarding response*. The level of confidence and satisfaction with each of the Lebanese government’s, the Ministry of Public Health’s, and health services’ response to the pandemic were measured; these 6 items’ responses were based on a 5-level Likert scale, with higher scores indicating more confidence and satisfaction and an answer option for having no opinion.

#### Covariates

Demographic and socioeconomic factors assessed included age (years), gender (male, female, other/prefer not to answer), marital status (married/engaged, single/widowed/divorced, prefer not to answer), nationality (Lebanese, non-Lebanese), educational attainment (≥ university degree or lower), being a current student status (yes/no), and employment status before the lockdown (employed which included: full-time employee, part-time employee, self-employed, student, retired, homemaker / unemployed). Given the potential impact on employment and income due to the economic crisis and the pandemic, participants self-reported any employment loss (yes/no) and change in income (stayed the same, decreased/stopped, increased) that occurred since the lockdown.

### Statistical analysis

Overall, 1,114 individuals accessed the survey, and 836 (75%) consented to participate. Of those 836, 658 had complete data on basic sociodemographic characteristics and 510 had complete data on the mental health measures; these 510 constituted our analytical sample (S1 Fig). We used a complete case approach in the analysis; some of later survey items (sources of information/trust and confidence/satisfaction with response to the pandemic) had some missing observations (missing rate <3.3%) and satisfaction/confidence items had response options as “no opinion” which were additionally coded as missing (missing rate for these specific items <6.3%; Table 1).

We first examined the associations of each of the binary outcome variables (indicating presence of elevated depressive and anxiety symptoms) with predictors of interest using chi-square tests for categorical and independent student t-test for continuous predictors. Results of these bivariate analyses are presented graphically in bar charts and detailed results of the tests are presented in S2–S5 Tables. The predictors investigated were demographic and socioeconomic factors, COVID-19-related worries (individual worries and composite worry score), general and specific daily life difficulties (each individual difficulty item and composite difficulty scores), support activities, levels of knowledge, sources of information, and trust in these sources, and reported confidence and satisfaction in governmental and health agencies’ responses. We then conducted logistic regressions to assess the relationship of these predictors with each of the binary mental health outcomes (results expressed as Odds Ratios (OR), 95% CI), adjusting for age, gender, change in income, and marital status. Associations remained comparable following further adjustment for education (university degree) and current student status. Two sociodemographic variables had “prefer not to answer” responses (n = 10 for gender and n = 3 for marital status) and these were coded as missing observations in the multivariable logistic regressions (sensitivity analysis keeping these response categories produced similar conclusions).

P-values<0.05 were considered statistically significant. Statistical analysis was conducted using Stata version 13 (StataCorp LP, College Station, TX, USA).

### Inclusivity in global research

Additional information regarding the ethical, cultural, and scientific considerations specific to inclusivity in global research is included in the Supporting Information.

## Results

### Sample characteristics

The samples’ mean age was 36.1 ± 11.3 years; 69.4% were women, 44.1% were married/engaged, 91.8% had a university degree or higher educational attainment and 95.1% were employed (Table 1). At the time of our survey, 2.9% had lost their job and 42.5% reported a decrease or loss of income since the start of the lockdown. Among participants, 32.3% had elevated depressive (PHQ-9 score ≥ 10) and 27.3% had elevated anxiety (GAD-7 score ≥10) symptoms (Table 1).

Compared to participants who initiated the survey but did not complete the mental health questionnaires, the analytical sample had higher educational attainment and lower proportion of married/engaged participants and was not different with regards to other demographic or socioeconomic indicators or reported COVID-19 worries (S1 Table).

### Distribution of outbreak/lockdown-related factors in total sample

The most frequently reported general difficulties encountered by participants during the lockdown were uncertainty regarding the future (74.51%), financial insecurity (52.16%), and emotional difficulties (42.16%); the most prevalent specific daily life difficulties encountered were work related (not being able to focus) (32.34%), having caregiving responsibilities (31.41%), and worrying about losing job (27.65%; Table 1). The main sources of support were family support (73.33%), having an outdoor space (68.43%), and friend support (60%).

The most prevalent outbreak-related worry was spreading the virus to others (80.20%), followed by worries of not getting proper care (35.10%) and isolation (32.75%). Participants had moderate to elevated knowledge about the pandemic with 84.04% reporting being knowledgeable/very knowledgeable about how to prevent the COVID-19 infection, 78.79% about COVID-19 symptoms, and 62.42% about the outbreak spread in the country; 45.25% reported seeing mostly conflicting facts about the virus. The most used sources for getting information on COVID-19 were regional/international health agencies (59.59%), local health agencies (Lebanese Ministry of Public Health) (54.34%), friends/family members (52.72%), and local media (52.53%); the least used source was healthcare professionals (35.76%). The most trusted sources of information were regional/international health agencies (68.42%), healthcare professionals (68.36%), local health agencies (Lebanese Ministry of Public Health) (48.58%), and local media (27.33%; Table 1). Overall, majority of people had low confidence with the pandemic response; with the highest confidence and satisfaction reported for the Ministry of Public health response (43.13% and 51.97% respectively; Table 1).

### Factors associated with elevated depressive and anxiety symptoms

**Demographic and socioeconomic factors.** Younger, student, and unemployed participants had significantly higher levels of mental health symptoms. Elevated depressive symptoms were also significantly higher among single participants (p<0.001) and those who had lower educational levels (p = 0.012), lower income before the lockdown (p = 0.001), and a recent job loss (p = 0.020; Table 2).

**Table 2:** Descriptive statistics of the sample with complete PHQ-9 and GAD-7 information.

	Depression scores			Anxiety scores		
	Low	High	p-value	Low	High	p-value
**Age**	38.43	31.26	**<0.001**	37.42	32.63	**<0.001**
**Gender**						
Male	107 (31.01%)	39 (23.64%)	0.130	116 (31.27%)	30 (21.58%)	0.076
Female	233 (67.54%)	121 (73.33%)		249 (67.11%)	105 (75.54%)	
Other/prefer not to answer	5 (1.45%)	5 (3.03%)		6 (1.62%)	4 (2.88%)	
**Marital Status**						
Married/Engaged	176 (51.01%)	49 (29.70%)	**<0.001**	172 (46.36%)	53 (38.13%)	0.242
Single/Widow/Divorced	166 (48.12%)	115 (69.70%)		196 (52.83%)	85 (61.15%)	
Prefer not to answer	3 (0.87%)	1 (0.60%)		3 (0.81%)	1 (0.72%)	
**University degree**						
Yes	324 (93.91%)	144 (87.27%)	**0.012**	344 (92.72%)	124 (89.21%)	0.307
No	17 (4.93%)	20 (12.12%)		23 (6.20%)	14 (10.07%)	
Prefer not to answer	4 (1.16%)	1 (0.61%)		4 (1.08%)	1 (0.72%)	
**Lebanese**						
Yes	330 (95.65%)	155 (93.94%)	0.704	355 (95.69%)	130 (93.53%)	0.402
No	12 (3.48%)	8 (4.85%)		12 (3.23%)	8 (5.75%)	
Prefer not to answer	3 (0.87%)	2 (1.21%)		4 (1.08%)	1 (0.72%)	
**Current students**						
Yes	38 (11%)	41 (24.8%)	**<0.001**	49 (13.2%)	30 (21.6%)	**0.020**
No	307 (89%)	124 (75.2%)		322 (86.8%)	109 (78.4%)	
**Employment status**						
Employed**(**Full-time,part-time,self-employed,retired,homemaker,student)	335 (97.10%)	150 (90.91%)	**0.002**	359 (96.77%)	126 (90.65%)	**0.004**
Unemployed (unemployed and seeking work, unemployed and not seeking work)	10 (2.90%)	15 (9.09%)		12 (3.23%)	13 (9.35%)	
**Change in employment since lockdown**						
*Loss of Job*						
Yes	6 (1.7%)	9 (5.5%)	**0.020**	10 (2.7%)	5(3.6%)	0.592
No	339 (98.3%)	156 (94.5%)		361 (97.3%)	134 (96.4%)	
**Income Before lockdown**						
Lower category (0–2,000,000 L.L)	47 (13.62%)	43 (26.06%)	**0.001**	59 (15.90%)	31 (22.30%)	0.185
Higher category (>2,000,000 L.L)	266 (77.10%)	104 (63.03%)		277 (74.66%)	93 (66.91%)	
Not sure/prefer not to answer	32 (9.28%)	18 (10.91%)		35 (9.44%)	15 (10.79%)	
**Change in Income since lockdown**						
No income before/after	52 (15.1%)	40 (24.2%)	**0.029**	59 (15.9%)	33 (23.7%)	0.119
Stopped/Decreased	148 (42.9%)	69 (41.8%)		163 (43.9%)	54 (38.8%)	
Stayed the same/Increased	145 (42.0%)	56 (33.9%)		149 (40.2%)	52 (37.4%)	

Associations of each of the binary outcome variables (high/low depressive and anxiety symptoms) with predictors of interest were examined using chi-square tests for categorical and independent student t-test for continuous predictors.

#### General and specific daily life difficulties

**Fig 1:**
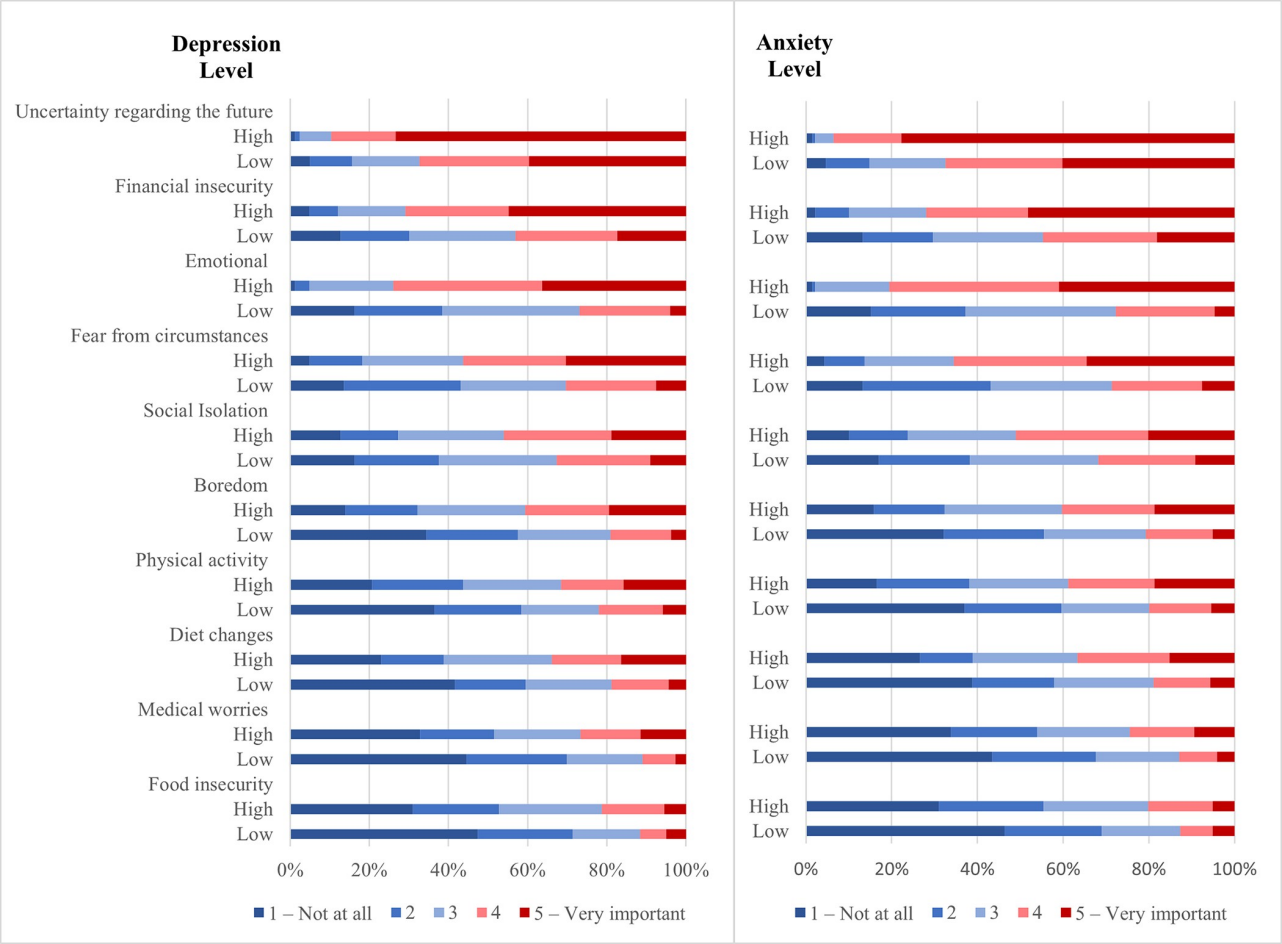
Reported general difficulties experienced during the lockdown in participants with and without elevated depression and anxiety scores.

Depressive and anxiety symptoms were consistently associated with significantly higher levels of *of each item of the general difficulties investigated* (p≤0.016) (Fig 1; S2 Table) and a higher number of difficulties was associated with higher odds of elevated depressive and anxiety symptoms, adjusted for age, gender, change in income, and marital status (OR = 1.53, 95% CI 1.38 1.70 and OR = 1.63, 95% CI 1.46 1.82 respectively) (Table 3).

**Table 3:** Factors related to depressive and anxiety symptoms in the total sample.

	Depression (unadjusted)			Depression* (adjusted)		Anxiety (unadjusted)		Anxiety* (adjusted)	
	OR	95%CI	OR		95%CI	OR	95%CI	OR	95%CI
**Outbreak-related worries**									
** Total score**	**1.27**	**1.14 1.41**	**1.36**		**1.20 1.53**	**1.38**	**1.24 1.55**	**1.47**	**1.30 1.67**
Getting infected	**1.76**	**1.18 2.61**	**1.94**		**1.25 3.01**	**2.48**	**1.64 3.74**	**2.81**	**1.81 4.37**
Health complications	1.13	0.75 1.69	1.35		0.86 2.12	**1.76**	**1.17 2.67**	**2.10**	**1.34 3.27**
Spreading the virus to others	1.58	0.96 2.60	1.28		0.74 2.21	**1.94**	**1.11 3.36**	1.64	0.92 2.94
Isolation	1.04	0.70 1.54	1.24		0.80 1.92	1.27	0.85 1.92	1.39	0.90 2.15
Not being able to get proper care if infected	**1.93**	**1.32 2.83**	**2.14**		**1.40 3.27**	**2.44**	**1.63 3.64**	**2.66**	**1.74 4.07**
Financial consequences	**2.98**	**1.97 4.52**	**3.58**		**2.24 5.72**	**3.02**	**1.98 4.63**	**3.37**	**2.13 5.34**
Negative reaction from people	**2.46**	**1.60 3.78**	**2.98**		**1.84 4.83**	**2.10**	**1.35 3.28**	**2.38**	**1.49 3.81**
**Difficulties**									
** Difficulties-general**	**1.52**	**1.38 1.68**	**1.53**		**1.38 1.70**	**1.64**	**1.47 1.83**	**1.63**	**1.46 1.82**
** Difficulties-specific**	**8.84**	**4.62 16.90**	**9.40**		**4.59 19.24**	**7.84**	**4.08 15.06**	**8.24**	**4.05 16.77**
**Support network**	0.92	0.85 1.00	0.93		0.85 1.02	**0.89**	**0.81 0.97**	**0.89**	**0.81 0.97**
**Knowledge about the pandemic (≥very knowledgeable) **									
knowledge about symptoms	**0.57**	**0.37 0.89**	**0.56**		**0.34 0.92**	0.70	0.44 1.11	0.69	0.42 1.14
Knowledge about prevention	0.67	0.41 1.09	0.64		0.50 1.16	0.68	0.41 1.14	0.64	0.37 1.12
Knowledge about spread	**0.68**	**0.46 0.99**	0.76		0.37 1.12	**0.55**	**0.37 0.83**	**0.56**	**0.37 0.86**
**Confidence with response to the pandemic (≥very confident)**									
Government	**0.46**	**0.30 0.71**	**0.49**		**0.31 0.76**	**0.51**	**0.32 0.79**	**0.54**	**0.34 0.85**
Ministry of Public Health	**0.50**	**0.33 0.74**	**0.53**		**0.35 0.81**	**0.45**	**0.29 0.69**	**0.48**	**0.32 0.74**
Health institutions	**0.65**	**0.43 0.97**	0.71		0.46 1.10	0.71	0.46 1.09	0.76	0.49 1.18
**Satisfaction with response to the pandemic (≥very satisfied)**									
Government	**0.50**	**0.34 0.74**	**0.51**		**0.33 0.77**	**0.51**	**0.34 0.77**	**0.53**	**0.34 0.81**
Ministry of Public Health	**0.41**	**0.28 0.61**	**0.43**		**0.28 0.65**	**0.36**	**0.24 0.55**	**0.36**	**0.24 0.56**
Health institutions	**0.52**	**0.35 0.77**	**0.55**		**0.37 0.83**	**0.53**	**0.35 0.79**	**0.54**	**0.36 0.82**
**Sources of information (≥more than half the time) **									
Health agencies (local: ministry of health)	1.12	0.76 1.63	1.04		0.69 1.57	**0.67**	**0.45 0.99**	**0.59**	**0.39 0.89**
Health agencies (International: WHO, CDC)	1.39	0.94 2.04	1.27		0.83 1.94	1.41	0.93 2.12	1.29	0.84 1.98
Health professionals	0.82	0.55 1.22	0.85		0.55 1.30	1.11	0.74 1.67	1.18	0.77 1.80
Public opinion	**1.88**	**1.28 2.76**	1.57		1.03 2.38	**1.55**	**1.04 2.31**	1.34	0.88 2.05
People you talk to daily	1.37	0.94 2.01	1.25		0.82 1.89	1.10	0.74 1.63	1.02	0.67 1.56
Media	1.07	0.74 1.56	1.11		0.74 1.67	0.91	0.61 1.34	0.89	0.59 1.34
**Trust in information (≥very trustworthy)**									
Health agencies (local: ministry of health)	**0.58**	**0.39 0.85**	**0.48**		**0.31 0.73**	**0.47**	**0.31 0.71**	**0.42**	**0.27 0.65**
Health agencies (International: WHO, CDC)	1.25	0.83 1.89	1.04		0.65 1.65	1.00	0.65 1.52	0.91	0.57 1.44
Health professionals	0.74	0.50 1.11	**0.58**		**0.37 0.90**	0.78	0.51 1.19	0.70	0.44 1.09
Public opinion	**2.33**	**1.01 5.41**	2.21		0.91 5.35	1.74	0.73 4.12	1.63	0.68 3.94
People you talk to daily	0.88	0.56 1.36	0.90		0.56 1.44	1.10	0.70 1.73	1.14	0.71 1.81
Media	0.78	0.51 1.20	0.74		0.46 1.18	0.85	0.54 1.33	0.86	0.54 1.37
**Seen conflicting information across sources**	**1.48**	**1.02 2.16**	**1.73**		**1.14 2.61**	1.20	0.81 1.78	1.34	0.88 2.02

OR: odds ratio, CI: confidence interval

logistic regressions taking depressive and anxiety symptoms (binary mental health outcomes) as dependent variables and predictors including demographic and socioeconomic factors, COVID-19-related worries (individual worries and composite worry score), general and specific daily life difficulties (individual difficulties and composite difficulty scores), support activities, levels of knowledge, sources of information, and trust in these sources, and reported confidence and satisfaction in governmental and health agencies’ responses as independent variables.

*adjusted for age, gender, change in income, and marital status.

**Fig 2:**
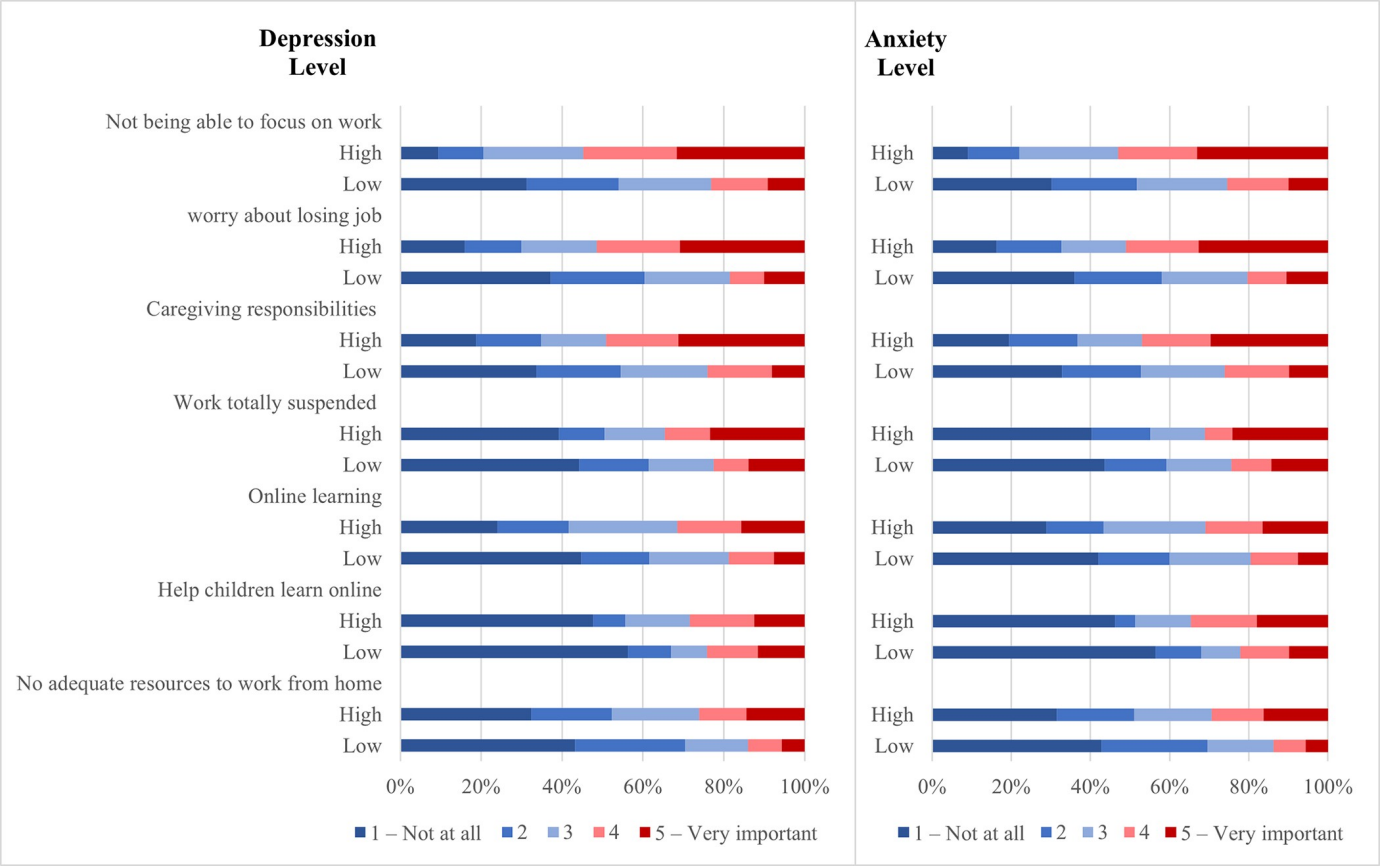
Reported specific daily life difficulties experienced during the lockdown in participants with and without elevated depression and anxiety scores.

Similarly, participants with higher depressive and anxiety symptoms had more difficulties with almost all specific daily life functioning items (Fig 2, S2 Table), and the differences were of large magnitude. For example, participants with elevated depressive and anxiety symptoms had more than twice higher prevalence of the top 3 reported difficulties: 49.2% vs 19.5% prevalence of work difficulties (inability to focus) for higher and lower depression groups and 40.8% vs 17.3% for higher and lower anxiety groups; 51.4% vs 18.6% prevalence of worry about losing employment for depression groups and 43.9% vs 16.1% for anxiety groups, and 45.8% vs 21.8% prevalence of difficulties with caregiving responsibilities for higher and lower depression groups and 38.3% vs 19.8% for anxiety groups ((p<0.001 for all abovementioned comparisons), Fig 2; S2 Table). Composite scores of specific daily functioning difficulties were associated with higher odds of elevated depression and anxiety symptoms in adjusted logistic regression (OR = 9.40, 95% CI 4.59 19.24 and OR = 8.24, 95% CI 4.05 16.77, respectively) (Table 3).

**Fig 3:**
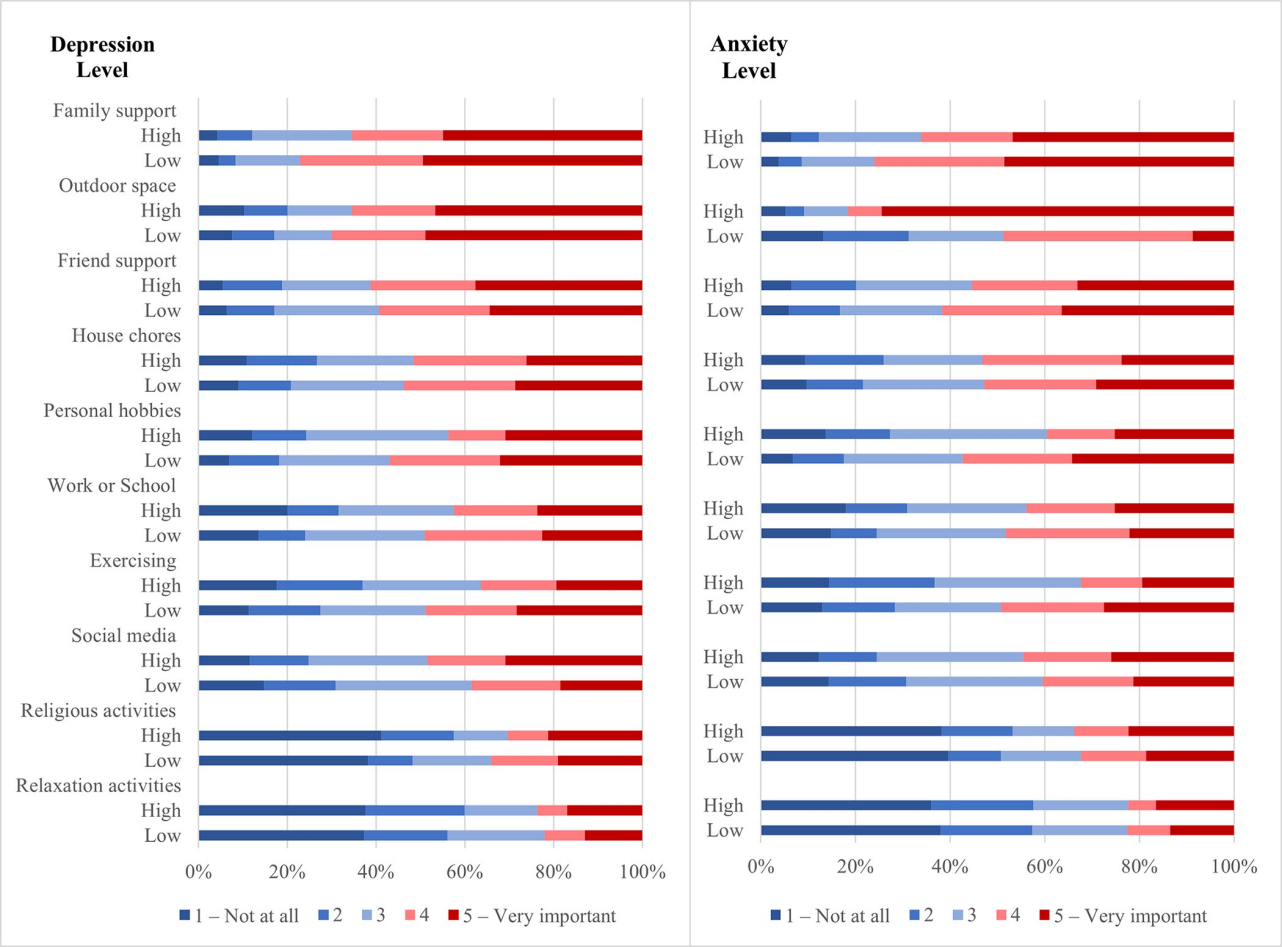
Sources of support during the lockdown in participants with and without elevated depression and anxiety scores.

**Sources of support.** Sources of support were different among participants with and without elevated mental health symptoms: Having an outdoor space and exercising/playing sports were related to lower anxiety symptoms (p = 0.024 and p = 0.013 respectively) and family support and religious activities were related to lower depressive symptoms (p = 0.03 and p = 0.055; Fig 3; S3 Table).

Participants with elevated depressive symptoms rated social media as a significantly more important source of support compared to those with lower symptoms (30.9% vs 18.5%, p = 0.040) (Fig 3, S3 Table**)**. Personal hobbies were an important source of support associated with lower depressive and anxiety symptoms (p = 0.008 and p = 0.004, respectively).

Higher overall support scores were significantly associated with lower odds of anxiety symptoms (adjusted OR = 0.89, 95% CI 0.81 0.97; Table 3).

#### Outbreak-related worries

Participants with elevated anxiety symptoms had significantly higher worries regarding all outbreak-related item (S2 Fig; S4 Table) and those with higher depressive symptoms had higher worry levels on most items (5 out of the 7 COVID-19-related worries S2 Fig; S4 Table). In adjusted analyses, higher COVID-related worries were significantly associated with higher odds of elevated depressive and anxiety symptoms (OR = 1.36, 95% CI = 1.20 1.53 and 1.47, 95%CI = 1.30 1.67, respectively; Table 3).

#### Level of knowledge about the pandemic

Lower knowledge about symptoms and exposure to conflicting information about COVID-19 were significantly associated with elevated depressive symptoms (adjusted OR = 0.56, 95% CI 0.34 0.92 and 1.73, 95% CI 1.14 2.61, respectively; Table 3); lower knowledge about the outbreak spread was significantly associated with elevated anxiety symptoms (adjusted OR = 0.56, 95% CI 0.37 0.86).

#### Sources of information and related trust

Specific sources of COVID-19 information were associated differently with mental health: participants who relied extensively on public opinion showed significantly higher levels of depressive and anxiety symptoms (p = 0.001 and p = 0.032 respectively) whereas those who often used local health agencies as their source of information (Ministry of Public Health) had significantly lower levels of anxiety (p = 0.045; S5 Table).

Regarding trust, higher trust in the information provided by local health agencies were related to lower depressive and anxiety symptoms (p = 0.005 and p<0.001, respectively), whereas higher trust in public opinion was significantly related to higher depressive symptoms (p = 0.043; S5 Table).

Adjusted analysis further supported the link between use and trust in the information provided by local health agencies and lower mental health symptoms (Table 3). Additionally, trust in the information received from healthcare professionals was related to lower depressive symptoms (adjusted OR = 0.58, 95% CI 0.37 0.90; Table 3).

#### Satisfaction and confidence with response to the pandemic

There was a consistent pattern of lower satisfaction and confidence regarding the response of all entities (government, Ministry of Public Health, and local health services) being significantly related to higher levels of depressive and anxiety symptoms (S3 Fig; Table 3)–confidence in health institutions was not significantly related to mental health symptoms in adjusted analysis.

Repeated analysis for Table 3 with further adjustment for education (university degree) and current student status yielded comparable conclusions (S6 Table).

## Discussion

### Main findings

This study showed that the mental health burden was substantial during the first COVID-19 lockdown in Lebanon and that people faced high levels of emotional, financial, and daily life difficulties adapting to the lockdown circumstances, with difficulties being significantly exacerbated among those with depressive and anxiety symptoms. Mental health symptoms were associated with all key aspects of the pandemic and response measures. Having higher outbreak-related worries, lower knowledge about the pandemic, lower reliance, trust, and satisfaction with the information and response provided by governmental and health institutions were associated with higher depressive and anxiety symptoms. Results indicate that numerous sources of distress are at play during outbreaks and lockdowns and highlight key elements of the response measures that can simultaneously mitigate the pandemic and mental health burden, notably increasing the reach and trustworthiness of information dissminated and integrating emotional and financial support and protection of daily life functioning.

### Results in context

The prevalence of elevated depressive and anxiety symptoms was high in our sample, despite the low infection rates at the time in the country (0.6% had a COVID-19 diagnosis in the sample and the average national count of cases was ~0.1/100,000). Higher depression and anxiety prevalence has often been reported during the COVID-19 pandemic and prior outbreaks and quarantines of the Severe Acute Respiratory Syndrome (SARS) in 2003 and Middle East Respiratory Syndrome (MERS) in 2015 [8, 32–35]. We note that the prevalence of depressive (32.3%) and anxiety symptoms (27.3%) found in our sample were among the highest reported across countries, and consistent with estimates reported in other samples in Lebanon [36]. Indeed, except for one study conducted in the UK during the first lockdown, estimating a similar prevalence of depression (32%) [37], most other studies reported lower prevalence rates of depression of 27.9% in China [38], 26% in Poland [39], 24.7% in Italy [40],and 8.3% in Spain [41] and of anxiety (8.1% to 24.7%) [40, 41]. The elevated mental health burden observed in Lebanese samples may be explained by the additional challenges imposed by the severe economic crisis and sociopolitical turmoil that began in October 2019 [42]. Periods of economic recession and financial vulnerability are generally associated with increased mental health problems, especially in low- and middle-income countries, where resources, awareness, and intervention strategies are limited [43, 44]. Recent data also show that the pandemic exacerbated mental health burden among socio-economically disadvantaged groups [43], in line with our findings of several socioeconomic indicators (lower educational attainment, job loss, lower income before the pandemic, and worsening income because of the pandemic) being related to higher depressive symptoms. Further, financial insecurity and worries about job loss were among the top reported difficulties experienced during the lockdown in our sample and were related to poorer mental health. This is concordant with the results of a study conducted in the U.S., where financial concerns about the economic consequences of the pandemic and greater job insecurity were related to greater depressive and anxiety symptoms [45].

Besides the financial stressors, our results showed higher depressive and anxiety symptoms in younger single participants and students during the first lockdown period, in line with findings from other populations during the pandemic. Young adults and college students are known to be at a higher risk for mental health [38, 46–48], with mental health conditions accounting for almost half of the nonfatal burden of disease among people aged 10 to 25 years [49]. The insufficient readiness of the educational sector in Lebanon with its scarce resources and infrastructural limitations for online learning added enormous challenges to students in our context. This can further explain the high frequency of difficulties specific to learning reported by our participants, and which were negatively related to mental health. Therefore, supporting the mental health of young people in this pandemic and crisis setting is an important public health priority. Marital status has also been generally linked to higher depression and anxiety risk, with married individuals being at lower risk [15, 38]. In the pandemic and lockdown context, the higher depression and anxiety symptoms among unmarried individuals might further be reflecting increased social isolation, vulnerability, and lower daily social and logistical support during these difficult times. Finally, the presence of gender differences in mental health outcomes is widely recognized [12, 15, 50, 51]; women in our sample also had a higher proportion of elevated depression and anxiety symptoms (albeit not statistically significant).

Another important finding was that 3 of the 4 most frequently reported difficulties experienced during the lockdown were related to fear and emotional difficulties (uncertainty regarding the future, emotional difficulties, and fear of circumstances). This suggests a substantial and diffuse emotional burden, especially earlier in the pandemic when a lot was still unknown regarding the COVID-19 illness and duration of the pandemic and lockdown measures. These difficulties were significantly higher among people with elevated depressive and anxiety symptoms. Similarly, a longitudinal study in Norway found that difficulties in the regulation of negative emotions and interpersonal problems related to the outbreak were associated with anxiety and depression throughout the pandemic and beyond [52]. Such results warrant more investigations and recognition of the widespread emotional toll related to the pandemic and lockdown measures, which can inform efforts to sustain the well-being of the general population and to identify the contexts where exacerbated mental health symptoms are emerging.

#### Sources of support

One important protective factor was personal hobbies, which was related to both lower anxiety and depressive symptoms during the lockdown. Our results also highlight the role of a wide spectrum of factors that may alleviate mental health challenges (exercising, family support, religious activities, limited engagement with social media, and having an outdoor space). Several studies have found similar results concerning coping strategies in relation to mental health. In Ireland, exercising, pursuing hobbies, and being outdoors were associated with the greatest affective benefits during the pandemic [53]. A Spanish study also showed that pursuing hobbies, and staying outdoors or looking outside were the best predictors of lower levels of depressive symptoms, and not reading news/updates about COVID-19 frequently were the best predictors of lower levels of anxiety symptoms [54]. Family support was associated with mitigating depressive symptoms in an Italian sample [55]. Furthermore, a systematic review on COVID-19 and mental health showed that active social media use was negatively related to depression and a predictor of secondary trauma [56]. In addition, positive religious coping was associated with reduced depressive symptoms in Malaysian healthcare workers and in American Orthodox Jewish populations [57, 58].

We note that in these aforementioned countries, stimulus or livelihood support strategies were implemented which undoubtedly helped sustain personal activities [59, 60]. In Lebanon, no support strategies were implemented because of the parallel economic crisis. Our and other studies’ results highlight the importance of sustaining and encouraging personal hobbies during lockdowns and in devising lockdown responses that can safeguard these positive activities and ensure they are accessible to all segments of the community.

#### Pandemic and pandemic-response factors

We found negative associations between all studied COVID-19-related worries and mental health; these associations were of large magnitude, especially for worries about financial consequences, stigma, health complications and accessing proper healthcare, getting infected, and spreading the virus to others. Earlier Swedish and Norwegian studies reported that being worried about spreading the infection to close ones was a main predictor of mental distress during the early phase of the COVID-19 pandemic [61, 62]. Fear of self-infection or having an infected family member had the strongest association with increased levels of anxiety and depression in other studies conducted in China and Italy [32, 63]. COVID-19 discrimination and stigma were also shown to have significant burden on the mental health of individuals in Sweden and China [61, 64]. Our results further shed light on worries about health complications, healthcare access, and financial consequences, highlighting their importance in a context of socioeconomic precarity.

Consistent with previous studies, higher knowledge about the virus in our sample was associated with lower risk of elevated anxiety and depressive symptoms [65, 66]. After health agencies, friends/family members, and local media were the most used sources of COVID-19 information by our participants; however, the most trusted sources were health agencies and healthcare professionals, suggesting an important gap in acquiring trusted information. Importantly, sources of COVID-19 information and their trustworthiness were closely linked to mental health. The use of local health agencies for information and higher trust in information disseminated by these agencies and healthcare professionals were associated with lower mental health symptoms; whereas higher reliance on and trusting public opinions had a negative association trend with depressive symptoms. Finally, exposure to conflicting information about COVID-19 was substantial and was also associated with higher depressive symptoms in this study. Throughout the pandemic, there was an elevated level of misinformation, and it was shown to exacerbate depressive symptoms in different populations [50, 51, 67].

Our findings also showed a clear pattern that lower confidence and satisfaction with the different institutions handling the pandemic were associated with poorer mental health outcomes. We note that, in our sample, the majority of participants reported trust and satisfaction with the government’s first response—despite the sociopolitical turmoil which was plaguing the country since October 2019—suggesting that participants may have separated their evaluation of response measures from the general sociopolitical climate. The association between negative mental health outcomes and lack of trust and satisfaction in governmental response has also been reported in a study surveying participants from the Netherlands, Greece, Germany, and the USA [68], and in two global studies each involving over 58 countries and105,000 participants [69, 70].

In line with emerging recommendations [71], our results suggest the need to expand the reach and access to information from healthcare professionals and healthcare agencies, especially in contexts similar to ours, where sociopolitical climate is largely negative. In parallel, results strongly advocate for increasing the accuracy of information shared on widely used sources such as local and social media, to broaden access to reliable information. These steps require active and efficient involvement of governmental and health agencies and the implementation of strategies that can simultaneously address multiple identified challenges. For example, increasing access to reliable and valid information about the pandemic through social media can be an important resource to alleviate both uncertainty and mental health in general, and in younger individuals in particular, as they are typically more engaged with social media. Social media platforms can also be used to increase awareness about mental health difficulties and to increase access of young people to mental health care through targeted online mental health interventions.

### Strengths and limitations

This study had several limitations. The cross-sectional design makes it difficult to draw conclusions about the directionality of associations and reverse causality is possible (i.e., that mental health distress was not a consequence of the outbreak and lockdown but preexisting risk for mental health problems increased the challenges faced during the pandemic). From a public health perspective, both directions of this association are important and our results show the widespread connections between mental health symptoms and several facets of the outbreak and consequent response measures. In addition, any delayed impact on mental health cannot be captured as the survey was conducted after the first lockdown. While an online survey distributed via the different social media platforms was deemed the safest and most efficient strategy to reach a larger number of participants during lockdown, it added several limitations in the representativeness of the sample (restricting the inclusion of people with limited internet access and social medial use and those with lower educational and digital literacy levels). Mental health challenges and difficulties may be more prevalent in a more representative sample that includes higher proportion of disadvantaged and vulnerable communities. Moreover, some associations (such as the ones of socioeconomic indicators and pandemic-related knowledge with mental health symptoms) may be more prominent in larger and more diverse samples.

## Conclusion

Our study shows that mental health burden among Lebanese adults during the first COVID-19 lockdown was among the highest reported worldwide, especially among university students and young adults. People with elevated depressive and anxiety symptoms had an important clustering of negative experiences, including individual emotional and functional challenges, more pandemic-related worries, poorer pandemic-related knowledge and means of information, and lower trust and satisfaction with the response measures implemented. Results also highlight a widespread toll of outbreak and lockdown measures and the need for prevention and intervention strategies that can alleviate both emotional and financial distress and improve access to health information and support. Results also strengthen the rationale for research and intervention efforts that can monitor and lessen the ramifications of this high mental health burden in the Lebanese population in these difficult times.

## Supporting information

S1 Table Comparison of the main socio-demographic variables between those who consented and began the survey and the final analytical sample (who completed the mental health components in the survey). (DOCX)

S2 Table General and specific difficulties during lockdown by depressive and anxiety symptoms scores. (DOCX)

S3 Table Activities and support networks in participants with complete PHQ-9 and GAD-7 information. (DOCX)

S4 Table Outbreak-related worries during lockdown in respondents with complete PHQ-9 and GAD-7 information. (DOCX)

S5 Table General knowledge and perceptions of trust and mental health during the lockdown with complete PHQ-9 and GAD-7 information. (DOCX)

S6 Table Factors related to depressive and anxiety symptoms in the total sample. (DOCX)

S1 Fig Flow chart for the selection of participants. (PDF)

S2 Fig Distribution of COVID-19-related worries in participants with and without elevated depression and anxiety scores. (PDF)

S3 Fig Confidence and Satisfaction with the different sector’s handling of the pandemic in participants with and without elevated depression and anxiety scores. (PDF)
