# Supplementary material for: Mental health during the COVID-19 pandemic and first lockdown in Lebanon: Risk factors and daily life difficulties in a multiple-crises setting
Source: PLoS One. 2024 Feb 16;19(2):e0297670. doi: 10.1371/journal.pone.0297670 (PMC10871500; doi:10.1371/journal.pone.0297670)
Supplement: S1 Table — (DOCX) [file pone.0297670.s001.docx]

**S1 Table.** Comparison of the main socio-demographic variables between those who consented and began the survey and the final analytical sample (who completed the mental health components in the survey).

|  | **Completed mental health components in the survey (N=510)** | | **Completed each of the following variables in the survey** | | | **p-value** |
| --- | --- | --- | --- | --- | --- | --- |
|  | **Mean or n** | **Sd or %** | **n responded to that variable but not others** | **Mean or n** | **Sd or %** |  |
| **Age** | 36.112 | 11.262 | 744 | 35.656 | 11.338 | 0.105 |
| **Gender** |  |  |  |  |  |  |
| Male | 146 | 28.63% | 744 | 214 | 28.76% | 0.401 |
| Female | 354 | 69.41% |  | 519 | 69.76% |  |
| Other/prefer not to answer | 10 | 1.96% |  | 11 | 1.48% |  |
| **Marital status** |  |  |  |  |  |  |
| Single/widow/divorced | 281 | 55.10% | 744 | 393 | 52.83% | **0.041** |
| Engaged/married | 225 | 44.12% |  | 341 | 45.83% |  |
| Prefer not to answer | 4 | 0.78% |  | 10 | 1.34% |  |
| **University Degree** |  |  |  |  |  |  |
| Yes | 468 | 91.76% | 744 | 670 | 90.05% | **0.048** |
| No | 37 | 7.25% |  | 67 | 9.01% |  |
| Prefer not to answer | 5 | 0.98% |  | 7 | 0.94% |  |
| **Current Student** |  |  |  |  |  |  |
| Yes* | 79 | 15.49% | 659 | 104 | 15.78% | 0.704 |
| No | 431 | 84.51% |  | 555 | 84.22% |  |
| **Employment Status** |  |  |  |  |  |  |
| Employed (full-time, part-time, self-employed)/retired/homemaker | 433 | 84.90% | 659 | 560 | 84.98% | 0.920 |
| Unemployed | 77 | 15.10% |  | 99 | 15.02% |  |
| **Employment change** |  |  |  |  |  |  |
| Loss of job |  |  |  |  |  |  |
| Yes | 15 | 2.94% | 659 | 23 | 3.49% | 0.155 |
| No | 495 | 97.06% |  | 636 | 96.51% |  |
| **Income Since lockdown** |  |  |  |  |  |  |
| No income before/after | 92 | 18.04% | 658 | 116 | 17.63% | 0.856 |
| Stopped/decreased | 217 | 42.55% |  | 280 | 42.55% |  |
| Stayed the same/increase | 201 | 39.41% |  | 262 | 39.82% |  |
| **Outbreak-related worries** |  |  | 616 |  |  |  |
| *Total worries score* | 2.537 | 1.780 |  | 2.538 | 1.801 | 0.998 |
| Getting infected | 150 | 29.41% |  | 176 | 28.57% | 0.311 |
| Health complications | 149 | 29.22% |  | 184 | 29.87% | 0.436 |
| Spreading the virus to others | 409 | 80.20% |  | 490 | 79.55% | 0.380 |
| Isolation | 167 | 32.75% |  | 199 | 32.31% | 0.609 |
| Not being able to get proper care if infected | 179 | 35.10% |  | 213 | 34.58% | 0.552 |
| Financial consequences | 129 | 25.29% |  | 162 | 26.30% | 0.214 |
| Negative reaction from people | 111 | 21.76% |  | 139 | 22.56% | 0.297 |

*38.30% of current students were also employed.
