# Supplementary material for: Mental health during the COVID-19 pandemic and first lockdown in Lebanon: Risk factors and daily life difficulties in a multiple-crises setting
Source: PLoS One. 2024 Feb 16;19(2):e0297670. doi: 10.1371/journal.pone.0297670 (PMC10871500; doi:10.1371/journal.pone.0297670)
Supplement: S2 Table — (DOCX) [file pone.0297670.s002.docx]

**S2 Table.** General and specific difficulties during lockdown by depressive and anxiety symptoms scores.

|  | | **PHQ-9** | | | **GAD-7** | | |
| --- | --- | --- | --- | --- | --- | --- | --- |
|  |  | **Score<10** | **Score≥10** | **P-value** | **Score<10** | **Score≥10** | **P-value** |
| ***General difficulties*** | | | | | | | |
| Emotional (e.g., feeling sad, stressed, demotivated) | Low | 252 (85.42%) | 43 (14.58%) | **<0.001** | 268 (90.85%) | 27 (9.15%) | **<0.001** |
|  | High | 93 (43.26%) | 122 (56.74%) |  | 103 (47.91%) | 112 (52.09%) |  |
| Fear due to the circumstances | Low | 241 (77.00%) | 72 (23.00%) | **<0.001** | 265 (84.66%) | 48 (15.34%) | **<0.001** |
|  | High | 104 (52.79%) | 93 (47.21%) |  | 106 (53.81%) | 91 (46.19%) |  |
| Social Isolation (not being able to see family and friends) | Low | 232 (72.27%) | 89 (27.73%) | **0.004** | 253 (78.82%) | 68 (21.18%) | **<0.001** |
|  | High | 113 (59.79%) | 76 (40.21%) |  | 118 (62.43%) | 71 (37.57%) |  |
| Boredom | Low | 279 (74.01%) | 98 (25.99%) | **<0.001** | 294 (77.98%) | 83 (22.02%) | **<0.001** |
|  | High | 66 (49.62%) | 67 (50.38%) |  | 77 (57.89%) | 56 (42.11%) |  |
| Financial insecurity and worries about your financial situation | Low | 196 (80.33%) | 48 (19.67%) | **<0.001** | 205 (84.02%) | 39 (15.98%) | **<0.001** |
|  | High | 149 (56.02%) | 117 (43.98%) |  | 166 (62.41%) | 100 (37.59%) |  |
| Food insecurity (not able to get basic food, water, and hygiene  items) | Low | 305 (70.11%) | 130 (29.89%) | **0.004** | 324 (74.48%) | 111 (25.52%) | **0.034** |
|  | High | 40 (53.33%) | 35 (46.67%) |  | 47 (62.67%) | 28 (37.33%) |  |
| Medical worries (unable to get regular medical care and  prescription) | Low | 307 (71.73%) | 121 (28.27%) | **<0.001** | 323 (75.74%) | 105 (24.53%) | **0.002** |
|  | High | 38 (46.34%) | 44 (53.66%) |  | 48 (58.54%) | 34 (41.46%) |  |
| Physical activity (unable to walk or do exercises) | Low | 269 (70.42%) | 113 (29.58%) | **0.021** | 297 (77.75%) | 85 (22.25%) | **<0.001** |
|  | High | 76 (59.38%) | 52 (40.63%) |  | 74 (57.81%) | 54 (42.19%) |  |
| Diet worries (experiencing diet changes) | Low | 280 (71.98%) | 109 (28.02%) | **<0.001** | 301 (77.38%) | 88 (22.62%) | **<0.001** |
|  | High | 65 (53.72%) | 56 (46.28%) |  | 70 (57.85%) | 51 (42.15%) |  |
| Uncertainty regarding the future | Low | 113 (86.92%) | 17 (13.08%) | **<0.001** | 121 (93.08%) | 9 (6.92%) | **<0.001** |
|  | High | 232 (61.05%) | 148 (38.95%) |  | 250 (65.79%) | 130 (34.21%) |  |
| **Total score -General** |  | 2.83 (2.17) | 4.91 (2.12) | **<0.001** | 2.85 (2.21) | 5.23 (1.84) | **<0.001** |
| ***Specific difficulties*** | | | | | | | |
| Work totally suspended | Low | 207 (74.73%) | 70 (25.27%) | **0.016** | 217 (78.34%) | 60 (21.66%) | 0.215 |
|  | High | 60 (61.86%) | 37 (38.14%) |  | 70 (72.16%) | 27 (27.84%) |  |
| Not having resources to work from home | Low | 227 (73.46%) | 82 (26.54%) | **0.005** | 244 (78.96%) | 65 (21.04%) | **0.001** |
|  | High | 37 (56.06%) | 29 (43.94%) |  | 39 (59.09%) | 27 (40.91%) |  |
| Not being able to focus on work | Low | 219 (80.51%) | 53 (19.49%) | **<0.001** | 225 (82.72%) | 47 (17.28%) | **<0.001** |
|  | High | 66 (50.77%) | 64 (49.23%) |  | 77 (59.23%) | 53 (40.77%) |  |
| Worry about job loss | Low | 228 (81.43%) | 52 (18.57%) | **<0.001** | 235 (83.93%) | 45 (16.07%) | **<0.001** |
|  | High | 52 (48.60%) | 55 (51.40%) |  | 60 (56.07%) | 47 (43.93%) |  |
| Caregiving responsibilities | Low | 205 (78.24%) | 57 (21.76%) | **<0.001** | 210 (80.15%) | 52 (19.85%) | **<0.001** |
|  | High | 65 (54.17%) | 55 (45.83%) |  | 74 (61.67%) | 46 (38.33%) |  |
| Learning Online | Low | 194 (72.39%) | 74 (27.61%) | **0.009** | 201 (75.00%) | 67 (25.00%) | **0.024** |
|  | High | 45 (56.96%) | 34 (43.04%) |  | 49 (62.03%) | 30 (37.97%) |  |
| Having to help children with online learning | Low | 163 (72.12%) | 63 (27.88%) | 0.443 | 175 (77.43%) | 51 (22.57%) | **0.030** |
|  | High | 52 (67.53%) | 25 (32.47%) |  | 50 (64.94%) | 27 (35.06%) |  |
| **Total score-specific difficulties** |  | 1.29 (1.36) | 2.55 (1.73) | **<0.001** | 1.31 (1.34) | 2.73 (1.78) | **<0.001** |
| **Average specific difficulties** |  | 0.08 (0.16) | 0.14 (0.23) | **0.0027** | 0.08 (0.16) | 0.15 (0.24) | **0.0009** |
| **Total** |  | 3.71 (2.87) | 7.61 (3.41) | **<0.001** | 3.81 (2.94) | 8.02 (3.24) | **<0.001** |
