# Supplementary material for: Mental health during the COVID-19 pandemic and first lockdown in Lebanon: Risk factors and daily life difficulties in a multiple-crises setting
Source: PLoS One. 2024 Feb 16;19(2):e0297670. doi: 10.1371/journal.pone.0297670 (PMC10871500; doi:10.1371/journal.pone.0297670)
Supplement: S3 Table — (DOCX) [file pone.0297670.s003.docx]

**S3 Table.** Activities and support networks in participants with complete PHQ-9 and GAD-7 information.

|  | | **PHQ-9** | | | **GAD-7** | | |
| --- | --- | --- | --- | --- | --- | --- | --- |
|  |  | **Score<10** | **Score≥10** | **P-value** | **Score<10** | **Score≥10** | **P-value** |
| **Family support** | 1 – Not at all | 16 (4.6%) | 7 (4.2%) | **0.03** | 14 (3.8%) | 9 (6.5%) | 0.149 |
|  | 2 | 13 (3.8%) | 13 (7.9%) |  | 18 (4.9%) | 8 (5.8%) |  |
|  | 3 | 50 (14.5%) | 37 (22.4%) |  | 57 (15.4%) | 30 (21.6%) |  |
|  | 4 | 96 (27.7%) | 34 (20.6%) |  | 102 (27.5%) | 27 (19.4%) |  |
|  | 5 – Very important | 171 (49.4%) | 74 (44.8%) |  | 180 (48.5%) | 65 (46.8%) |  |
| **Friend support** | 1 – Not at all | 22 (6.4%) | 9 (5.5%) | 0.757 | 22 (5.9%) | 9 (6.5%) | 0.766 |
|  | 2 | 37 (10.7%) | 22 (13.3%) |  | 40 (10.8%) | 19 (13.7%) |  |
|  | 3 | 82 (23.7%) | 33 (20.0%) |  | 80 (21.6%) | 34 (24.5%) |  |
|  | 4 | 86 (24.9%) | 39 (23.6%) |  | 94 (25.3%) | 31 (22.3%) |  |
|  | 5 – Very important | 119 (34.4%) | 62 (37.6%) |  | 135 (36.4%) | 46 (33.1%) |  |
| **Personal hobbies** | 1 – Not at all | 24 (6.9%) | 20 (12.1%) | **0.008** | 25 (6.7%) | 19 (13.7%) | **0.004** |
|  | 2 | 39 (11.3%) | 20 (12.1%) |  | 40 (10.8%) | 19 (13.7%) |  |
|  | 3 | 86 (24.9%) | 53 (32.1%) |  | 93 (25.1%) | 46 (33.1%) |  |
|  | 4 | 86 (24.9%) | 21 (12.7%) |  | 86 (23.2%) | 20 (14.4%) |  |
|  | 5 – Very important | 111 (32.1%) | 51 (30.9%) |  | 127 (34.2%) | 35 (25.2%) |  |
| **House chores (cooking, cleaning, tidying, ...)** | 1 – Not at all | 31 (9.0%) | 18 (10.9%) | 0.627 | 36 (9.7%) | 13 (9.4%) | 0.284 |
|  | 2 | 41 (11.8%) | 26 (15.8%) |  | 44 (11.9%) | 23 (16.5%) |  |
|  | 3 | 88 (25.4%) | 36 (21.8%) |  | 95 (25.6%) | 29 (20.9%) |  |
|  | 4 | 87 (25.1%) | 42 (25.5%) |  | 88 (23.7%) | 41 (29.5%) |  |
|  | 5 – Very important | 99 (28.6%) | 43 (26.1%) |  | 108 (29.1%) | 33 (23.7%) |  |
| **Exercising or playing sports** | 1 – Not at all | 39 (11.3%) | 29 (17.6%) | 0.071 | 48 (12.9%) | 20 (14.4%) | **0.013** |
|  | 2 | 56 (16.2%) | 32 (19.4%) |  | 57 (15.4%) | 31 (22.3%) |  |
|  | 3 | 82 (23.7%) | 44 (26.7%) |  | 83 (22.4%) | 43 (30.9%) |  |
|  | 4 | 71 (20.5%) | 28 (17.0%) |  | 81 (21.8%) | 18 (12.9%) |  |
|  | 5 – Very important | 98 (28.3%) | 32 (19.4%) |  | 102 (27.5%) | 27 (19.4%) |  |
| **Religious activities and praying** | 1 – Not at all | 132 (38.2%) | 68 (41.2%) | 0.055 | 147 (39.6%) | 53 (38.1%) | 0.473 |
|  | 2 | 35 (10.1%) | 27 (16.4%) |  | 41 (11.1%) | 21 (15.1%) |  |
|  | 3 | 61 (17.6%) | 20 (12.1%) |  | 63 (17.0%) | 18 (12.9%) |  |
|  | 4 | 52 (15.0%) | 15 (9.1%) |  | 51 (13.7%) | 16 (11.5%) |  |
|  | 5 – Very important | 66 (19.1%) | 35 (21.2%) |  | 69 (18.6%) | 31 (22.3%) |  |
| **Meditation, yoga, doing relaxation activities** | 1 – Not at all | 129 (37.3%) | 62 (37.6%) | 0.353 | 141 (38.0%) | 50 (38.0%) | 0.656 |
|  | 2 | 65 (18.8%) | 37 (22.4%) |  | 72 (19.4%) | 30 (21.6%) |  |
|  | 3 | 76 (22.0%) | 27 (16.4%) |  | 74 (19.9%) | 28 (20.1%) |  |
|  | 4 | 31 (9.0%) | 11 (6.7%) |  | 34 (9.2%) | 8 (5.8%) |  |
|  | 5 – Very important | 45 (13.0%) | 28 (17.0%) |  | 50 (13.5%) | 23 (16.5%) |  |
| **Work or School** | 1 – Not at all | 47 (13.6%) | 33 (20.0%) | 0.202 | 55 (14.8%) | 25 (18.0%) | 0.339 |
|  | 2 | 36 (10.4%) | 19 (11.5%) |  | 36 (9.7%) | 18 (12.9%) |  |
|  | 3 | 93 (26.9%) | 43 (26.1%) |  | 101 (27.2%) | 35 (25.2%) |  |
|  | 4 | 92 (26.6%) | 31 (18.8%) |  | 97 (26.1%) | 26 (18.7%) |  |
|  | 5 – Very important | 78 (22.5%) | 39 (23.6%) |  | 82 (22.1%) | 35 (25.2%) |  |
| **Having an outdoor space at home (balcony, garden, rooftop ...)** | 1 – Not at all | 26 (7.5%) | 17 (10.3%) | 0.804 | 27 (7.3%) | 16 (11.5%) | **0.024** |
|  | 2 | 33 (9.5%) | 16 (9.7%) |  | 37 (10.0%) | 12 (8.6%) |  |
|  | 3 | 45 (13.0%) | 24 (14.5%) |  | 41 (11.1%) | 28 (20.1%) |  |
|  | 4 | 73 (21.1%) | 31 (18.8%) |  | 82 (22.1%) | 22 (15.8%) |  |
|  | 5 – Very important | 169 (48.8%) | 77 (46.7%) |  | 184 (49.6%) | 61 (43.9%) |  |
| **Being on social media platforms (Instagram, Twitter, Facebook,**  **TikTok, etc.)** | 1 – Not at all | 51 (14.7%) | 19 (11.5%) | **0.040** | 53 (14.3%) | 17 (12.2%) | 0.630 |
|  | 2 | 56 (16.2%) | 22 (13.3%) |  | 61 (16.4%) | 17 (12.2%) |  |
|  | 3 | 106 (30.6%) | 44 (26.7%) |  | 107 (28.8%) | 43 (30.9%) |  |
|  | 4 | 69 (19.9%) | 29 (17.6%) |  | 71 (19.1%) | 26 (18.7%) |  |
|  | 5 – Very important | 64 (18.5%) | 51 (30.9%) |  | 79 (21.3%) | 36 (25.9%) |  |
| **Total score** |  | 5.09 (2.18) | 4.68 (2.51) | 0.0624 | 5.13 (2.27) | 4.51 (2.32) | **0.0069** |
