# Supplementary material for: Mental health during the COVID-19 pandemic and first lockdown in Lebanon: Risk factors and daily life difficulties in a multiple-crises setting
Source: PLoS One. 2024 Feb 16;19(2):e0297670. doi: 10.1371/journal.pone.0297670 (PMC10871500; doi:10.1371/journal.pone.0297670)
Supplement: S4 Table — (DOCX) [file pone.0297670.s004.docx]

**S4 Table.** Outbreak-related worries during lockdown in respondents with complete PHQ-9 and GAD-7 information.

|  | | **PHQ-9** | | | **GAD-7** | | |
| --- | --- | --- | --- | --- | --- | --- | --- |
|  |  | **Score<10**  **N=346** | **Score≥10**  **N=165** | **P-value** | **Score<10** | **Score≥10** | **P-value** |
| I am worried about contracting the coronavirus myself. | Not at all | 35 (10.1%) | 10 (6.1%) | **0.049** | 39 (10.5%) | 6 (4.3%) | **<0.001** |
|  | A little | 90 (26%) | 33 (20%) |  | 98 (26.4%) | 25 (18%) |  |
|  | Moderately | 132 (38.2%) | 60 (36.4%) |  | 145 (39.1%) | 47 (33.8%) |  |
|  | Very much | 64 (18.5%) | 44 (26.7%) |  | 67 (18.1%) | 41 (29.5%) |  |
|  | Extremely | 25 (7.2%) | 18 (10.9%) |  | 22 (5.9%) | 20 (14.4%) |  |
| I am worried about having health complications as a result of  contracting the virus. | Not at all | 44 (12.7%) | 26 (15.8%) | 0.234 | 51 (13.7%) | 19 (13.7%) | **0.009** |
|  | A little | 103 (29.8%) | 34 (20.6%) |  | 113 (30.5%) | 24 (17.3%) |  |
|  | Moderately | 101 (29.2%%) | 54 (32.7%) |  | 111 (29.9%) | 43 (30.9%) |  |
|  | Very much | 69 (19.9%%) | 33 (20.0%) |  | 69 (18.6%) | 33 (23.7%) |  |
|  | Extremely | 29 (8.4%%) | 18 (10.9%) |  | 27 (7.3%) | 20 (14.4%) |  |
| I am worried about spreading the virus to others if I contracted it  myself. | Not at all | 10 (2.9%) | 2 (1.2%) | 0.064 | 9 (2.4%) | 3 (2.2%) | **0.009** |
|  | A little | 21 (6.1%) | 9 (5.5%) |  | 26 (7.0%) | 4 (2.9%) |  |
|  | Moderately | 45 (13.0%) | 14 (8.5%) |  | 48 (12.9%) | 11 (7.9%) |  |
|  | Very much | 128 (37.0%) | 51 (30.9%) |  | 138 (37.2%) | 41 (29.5%) |  |
|  | Extremely | 142 (41.0%) | 89 (53.9%) |  | 150 (40.4%) | 80 (57.6%) |  |
| I am worried about being in isolation if I were to contract the  virus. | Not at all | 86 (24.9%) | 33 (20.0%) | 0.693 | 95 (25.6%) | 23 (16.5%) | 0.068 |
|  | A little | 74 (21.4%) | 34 (20.6%) |  | 82 (22.1%) | 26 (18.7%) |  |
|  | Moderately | 74 (21.4%) | 43 (26.1%) |  | 78 (21.0%) | 39 (28.1%) |  |
|  | Very much | 70 (20.2%) | 33 (20.0%) |  | 75 (20.2%) | 28 (20.1%) |  |
|  | Extremely | 42 (12.1%) | 22 (13.3%) |  | 41 (11.1%) | 23 (16.5%) |  |
| I am worried about not being able to get proper care and treatment  if I were to contract the virus. | Not at all | 67 (19.4%) | 27 (16.4%) | **0.005** | 77 (20.8%) | 17 (12.2%) | **<0.001** |
|  | A little | 71 (20.5%) | 21 (12.7%) |  | 74 (19.9%) | 18 (12.9%) |  |
|  | Moderately | 104 (30.1%) | 42 (25.5%) |  | 111 (29.9%) | 34 (24.5%) |  |
|  | Very much | 67 (19.4%) | 40 (24.2%) |  | 68 (18.3%) | 39 (28.1%) |  |
|  | Extremely | 37 (10.7%) | 35 (21.2%) |  | 41 (11.1%) | 31 (22.3%) |  |
| I am worried that if I contract the virus, it would have serious  financial consequences for me. | Not at all | 98 (28.3%) | 28 (17.0%) | **<0.001** | 104 (28.0%) | 22 (15.8%) | **<0.0001** |
|  | A little | 103 (29.8%) | 30 (18.2%) |  | 112 (30.2%) | 21 (15.1%) |  |
|  | Moderately | 81 (23.4%) | 41 (24.8%) |  | 84 (22.6%) | 38 (27.3%) |  |
|  | Very much | 44 (12.7%) | 40 (24.2%) |  | 51 (13.7%) | 32 (23.0%) |  |
|  | Extremely | 20 (5.8%) | 26 (15.8%) |  | 20 (5.4%) | 26 (18.7%) |  |
| I am worried that people will react negatively if they know I have  the infection. | Not at all | 128 (37.0%) | 43 (26.1%) | **0.001** | 135 (36.4%) | 36 (25.9%) | **0.004** |
|  | A little | 84 (24.3%) | 38 (23.0%) |  | 96 (25.9%) | 26 (18.7%) |  |
|  | Moderately | 76 (22.0%) | 30 (18.2%) |  | 73 (19.7%) | 33 (23.7%) |  |
|  | Very much | 33 (9.5%) | 29 (17.6%) |  | 38 (10.2%) | 23 (16.5%) |  |
|  | Extremely | 25 (7.2%) | 25 (15.2%) |  | 29 (7.8%) | 21 (15.1%) |  |
| **Total worries score** |  | 2.29 (1.75) | 3.05 (1.74) | **<0.001** | 2.25 (1.69) | 3.29 (1.79) | **<0.001** |
