# Supplementary material for: Mental health during the COVID-19 pandemic and first lockdown in Lebanon: Risk factors and daily life difficulties in a multiple-crises setting
Source: PLoS One. 2024 Feb 16;19(2):e0297670. doi: 10.1371/journal.pone.0297670 (PMC10871500; doi:10.1371/journal.pone.0297670)
Supplement: S5 Table — (DOCX) [file pone.0297670.s005.docx]

**S5 Table.** General knowledge and perceptions of trust and mental health during the lockdown with complete PHQ-9 and GAD-7 information.

|  | | **PHQ-9** | | | **GAD-7** | | |
| --- | --- | --- | --- | --- | --- | --- | --- |
|  |  | **Score<10** | **Score≥10** | **P-value** | **Score<10** | **Score≥10** | **P-value** |
| **Lockdown participation** | Low | 64 (75.29%) | 21 (24.71%) | 0.099 | 67 (78.82%) | 18 (21.18%) | 0.168 |
|  | High | 281 (66.12%) | 144 (33.88%) |  | 304 (71.53%) | 121 (28.47%) |  |
| **Knowledge about the pandemic** | | | | | | | |
| **Prevention** | Low | 47 (59.49%) | 32 (40.51%) | 0.108 | 52 (65.82%) | 27 (34.18%) | 0.145 |
|  | High | 286 (68.75%) | 130 (31.25%) |  | 307 (73.80%) | 109 (26.20%) |  |
| **Symptoms** | Low | 60 (57.14%) | 45 (42.86%) | **0.013** | 70 (66.67%) | 35 (33.33%) | 0.130 |
|  | High | 273 (70.00%) | 117 (30.00%) |  | 289 (74.10%) | 101 (25.90%) |  |
| **Spread in Lebanon** | Low | 115 (61.83%) | 71 (38.17%) | **0.045** | 121 (65.05%) | 65 (34.95%) | **0.004** |
|  | High | 218 (70.55%) | 91 (29.45%) |  | 238 (77.02%) | 71 (22.98%) |  |
| **Conflicting information** | Yes | 140 (62.50%) | 84 (37.50%) | **0.040** | 158 (70.54%) | 66 (29.46%) | 0.367 |
|  | No | 193 (71.22%) | 78 (28.78%) |  | 201 (74.17%) | 70 (25.83%) |  |
| **Sources of information** | | | | | | | |
| Health agencies (local: ministry of health) | Low | 155 (68.58%) | 71 (31.42%) | 0.569 | 154 (68.14%) | 72 (31.86%) | **0.045** |
|  | High | 178 (66.17%) | 91 (33.83%) |  | 205 (76.21%) | 64 (23.79%) |  |
| Health agencies (International: WHO, CDC) | Low | 143 (71.50%) | 57 (28.50%) | 0.099 | 153 (76.50%) | 47 (23.50%) | 0.103 |
|  | High | 190 (64.41%) | 105 (35.59%) |  | 206 (69.83%) | 89 (30.17%) |  |
| Health professionals | Low | 209 (65.72%) | 109 (34.28%) | 0.325 | 233 (73.27%) | 85 (26.73%) | 0.619 |
|  | High | 124 (70.06%) | 53 (29.94%) |  | 126 (71.19%) | 51 (28.81%) |  |
| Public opinion | Low | 223 (72.64%) | 84 (27.36%) | **0.001** | 233 (75.90%) | 74 (24.10%) | **0.032** |
|  | High | 110 (58.51%) | 78 (41.49%) |  | 126 (67.02%) | 62 (32.98%) |  |
| People you talk to daily | Low | 166 (70.94%) | 68 (29.06%) | 0.100 | 172 (73.50%) | 62 (26.50%) | 0.644 |
|  | High | 167 (63.98%) | 94 (36.02%) |  | 187 (71.65%) | 74 (28.35%) |  |
| Media | Low | 160 (68.09%) | 75 (31.91%) | 0.714 | 168 (71.49%) | 67 (28.51%) | 0.624 |
|  | High | 173 (66.54%) | 87 (33.46%) |  | 191 (73.46%) | 69 (26.54%) |  |
| **Trust in information** | | | | | | | |
| Health agencies (local: ministry of health) | Low | 156 (61.42%) | 98 (38.58%) | **0.005** | 166 (65.35%) | 88 (34.65%) | **<0.001** |
|  | High | 176 (73.33%) | 64 (26.67%) |  | 192 (80.00%) | 48 (20.00%) |  |
| Health agencies (International: WHO, CDC) | Low | 110 (70.51%) | 46 (29.49%) | 0.288 | 113 (72.44%) | 43 (27.56%) | 0.991 |
|  | High | 222 (65.68%) | 116(34.32%) |  | 245 (72.49%) | 93 (27.51%) |  |
| Health professionals | Low | 98 (62.82%) | 58 (37.18%) | 0.145 | 108 (69.23%) | 48 (30.77%) | 0.251 |
|  | High | 234 (69.44%) | 103 (30.56%) |  | 250 (74.18%) | 87 (25.82%) |  |
| Public opinion | Low | 321 (68.15%) | 150 (31.85%) | **0.043** | 344 (73.04%) | 127 (26.96%) | 0.202 |
|  | High | 11 (47.83%) | 12 (52.17%) |  | 14 (60.87%) | 9 (39.13%) |  |
| People you talk to daily | Low | 246 (66.49%) | 124 (33.51%) | 0.556 | 270 (72.97%) | 100 (27.03%) | 0.665 |
|  | High | 86 (69.35%) | 38 (30.65% |  | 88 (70.97%) | 36 (29.03%) |  |
| Media | Low | 236 (65.74%) | 123 (34.26%) | 0.257 | 257 (71.59%) | 102 (28.41%) | 0.474 |
|  | High | 96 (71.11%) | 39 (28.89%) |  | 101 (74.81%) | 34 (25.19%) |  |
| **Confidence with response to the pandemic** | | | | | | | |
| Government | Low | 185 (61.46%) | 116 (38.54%) | **<0.001** | 205 (68.11%) | 96 (31.89%) | **0.003** |
|  | High | 137 (77.40%) | 40 (22.60%) |  | 143 (80.79%) | 34 (19.21%) |  |
| Ministry of public health | Low | 165 (60.44%) | 108 (39.56%) | **0.001** | 178 (65.20%) | 95 (34.80%) | **<0.001** |
|  | High | 156 (75.36%) | 51 (24.64%) |  | 167 (80.68%) | 40 (19.32%) |  |
| Health institutions | Low | 196 (63.43%) | 113 (36.57%) | **0.036** | 215 (69.58%) | 94 (30.42%) | 0.115 |
|  | High | 126 (72.83%) | 47 (27.17%) |  | 132 (76.30%) | 41 (23.70%) |  |
| **Satisfaction with response to the pandemic** | | | | | | | |
| Government | Low | 151 (59.68%) | 102 (40.32%) | **<0.001** | 167 (66.01%) | 86 (33.99%) | **0.001** |
|  | High | 172 (74.78%) | 58 (25.22%) |  | 182 (79.13%) | 48 (20.87%) |  |
| Ministry of public health | Low | 132 (56.90%) | 100 (43.10%) | **<0.001** | 143 (61.64%) | 89 (38.36%) | **<0.001** |
|  | High | 191 (76.10%) | 60 (23.90%) |  | 205 (81.67%) | 46 (18.33%) |  |
| Health institutions | Low | 137 (59.31%) | 94 (40.69%) | **0.001** | 151 (65.37%) | 80 (34.63%) | **0.002** |
|  | High | 182 (73.68%) | 65 (26.32%) |  | 193 (78.13%) | 54 (21.86%) |  |
