# Supplementary material for: Mental health during the COVID-19 pandemic and first lockdown in Lebanon: Risk factors and daily life difficulties in a multiple-crises setting
Source: PLoS One. 2024 Feb 16;19(2):e0297670. doi: 10.1371/journal.pone.0297670 (PMC10871500; doi:10.1371/journal.pone.0297670)
Supplement: S6 Table — (DOCX) [file pone.0297670.s006.docx]

**S6 Table.** Factors related to depressive and anxiety symptoms in the total sample.

|  | Depression^⁋^ | | | Depression* | | Anxiety^⁋^ | | Anxiety* | |
| --- | --- | --- | --- | --- | --- | --- | --- | --- | --- |
|  | OR | 95%CI | OR | | 95%CI | OR | 95%CI | OR | 95%CI |
| Outbreak-related worries |  |  |  | |  |  | |  | |
| Total score | **1.33** | **1.79 1.51** | **1.36** | | **1.20 1.53** | **1.46** | **1.28 1.65** | **1.47** | **1.30 1.67** |
| Getting infected | **1.91** | **1.22 2.98** | **1.94** | | **1.25 3.01** | **2.81** | **1.80 4.38** | **2.81** | **1.81 4.37** |
| Health complications | 1.32 | 0.84 2.07 | 1.35 | | 0.86 2.12 | **2.08** | **1.33 3.25** | **2.10** | **1.34 3.27** |
| Spreading the virus to others | 1.18 | 0.68 2.06 | 1.28 | | 0.74 2.21 | 1.56 | 0.87 2.81 | 1.64 | 0.92 2.94 |
| Isolation | 1.19 | 0.77 1.85 | 1.24 | | 0.80 1.92 | 1.35 | 0.87 2.09 | 1.39 | 0.90 2.15 |
| Not being able to get proper care if infected | **2.03** | **1.32 3.12** | **2.14** | | **1.40 3.27** | **2.62** | **1.71 4.03** | **2.66** | **1.74 4.07** |
| Financial consequences | **3.42** | **2.13 5.49** | **3.58** | | **2.24 5.72** | **3.32** | **2.09 5.28** | **3.37** | **2.13 5.34** |
| Negative reaction from people | **2.89** | **1.77 4.70** | **2.98** | | **1.84 4.83** | **2.30** | **1.43 3.69** | **2.38** | **1.49 3.81** |
| Difficulties |  |  |  | |  |  |  |  |  |
| Difficulties-general | **1.53** | **1.38 1.70** | **1.53** | | **1.38 1.70** | **1.64** | **1.46 1.84** | **1.63** | **1.46 1.82** |
| Difficulties-specific | **9.69** | **4.67 20.12** | **9.40** | | **4.59 19.24** | **8.25** | **4.03 16.91** | **8.24** | **4.05 16.77** |
| Support network | 0.94 | 0.86 1.03 | 0.93 | | 0.85 1.02 | **0.90** | **0.82 0.98** | **0.89** | **0.81 0.97** |
| Knowledge about the pandemic (≥very knowledgeable) | | | | | | | | | |
| knowledge about symptoms | **0.59** | **0.35 0.98** | **0.56** | | **0.34 0.92** | 0.71 | 0.42 1.18 | 0.69 | 0.42 1.14 |
| Knowledge about prevention | 0.65 | 0.37 1.15 | 0.64 | | 0.50 1.16 | 0.63 | 0.36 1.10 | 0.64 | 0.37 1.12 |
| Knowledge about spread | 0.79 | 0.52 1.22 | 0.76 | | 0.37 1.12 | **0.56** | **0.37 0.86** | **0.56** | **0.37 0.86** |
| Confidence with response to the pandemic (≥very confident) | | | | | | | | | |
| Government | **0.46** | **0.29 0.74** | **0.49** | | **0.31 0.76** | **0.54** | **0.34 0.85** | **0.54** | **0.34 0.85** |
| Ministry of Public Health | **0.50** | **0.33 0.78** | **0.53** | | **0.35 0.81** | **0.47** | **0.30 0.73** | **0.48** | **0.32 0.74** |
| Health institutions | 0.68 | 0.44 1.06 | 0.71 | | 0.46 1.10 | 0.74 | 0.47 1.15 | 0.76 | 0.49 1.18 |
| Satisfaction with response to the pandemic (≥very satisfied) | | | | | | | | | |
| Government | **0.47** | **0.31 0.73** | **0.51** | | **0.33 0.77** | **0.51** | **0.33 0.78** | **0.53** | **0.34 0.81** |
| Ministry of Public Health | **0.40** | **0.26 0.62** | **0.43** | | **0.28 0.65** | **0.35** | **0.23 0.54** | **0.36** | **0.24 0.56** |
| Health institutions | **0.53** | **0.35 0.81** | **0.55** | | **0.37 0.83** | **0.53** | **0.35 0.81** | **0.54** | **0.36 0.82** |
| Sources of information (≥more than half the time) | | | | | | | | | |
| Health agencies (local: ministry of health) | 1.04 | 0.69 1.57 | 1.04 | | 0.69 1.57 | **0.58** | **0.38 0.88** | **0.59** | **0.39 0.89** |
| Health agencies (International: WHO, CDC) | 1.31 | 0.85 2.01 | 1.27 | | 0.83 1.94 | 1.29 | 0.84 1.99 | 1.29 | 0.84 1.98 |
| Health professionals | 0.87 | 0.57 1.34 | 0.85 | | 0.55 1.30 | 1.20 | 0.78 1.85 | 1.18 | 0.77 1.80 |
| Public opinion | **1.59** | **1.04 2.42** | **1.57** | | **1.03 2.38** | 1.34 | 0.87 2.05 | 1.34 | 0.88 2.05 |
| People you talk to daily | 1.24 | 0.81 1.89 | 1.25 | | 0.82 1.89 | 1.02 | 0.67 1.55 | 1.02 | 0.67 1.56 |
| Media | 1.06 | 0.70 1.60 | 1.11 | | 0.74 1.67 | 0.86 | 0.56 1.30 | 0.89 | 0.59 1.34 |
| Trust in information (≥very trustworthy) | | | | | | | | | |
| Health agencies (local: ministry of health) | **0.46** | **0.30 0.71** | **0.48** | | **0.31 0.73** | **0.41** | **0.26 0.63** | **0.42** | **0.27 0.65** |
| Health agencies (International: WHO, CDC) | 1.11 | 0.69 1.77 | 1.04 | | 0.65 1.65 | 0.93 | 0.58 1.48 | 0.91 | 0.57 1.44 |
| Health professionals | **0.57** | **0.36 0.90** | **0.58** | | **0.37 0.90** | 0.69 | 0.44 1.85 | 0.70 | 0.44 1.09 |
| Public opinion | 2.24 | 0.90 5.56 | 2.21 | | 0.91 5.35 | 1.75 | 0.71 4.28 | 1.63 | 0.68 3.94 |
| People you talk to daily | 0.88 | 0.55 1.42 | 0.90 | | 0.56 1.44 | 1.14 | 0.72 1.83 | 1.14 | 0.71 1.81 |
| Media | 0.71 | 0.44 1.14 | 0.74 | | 0.46 1.18 | 0.84 | 0.52 1.34 | 0.86 | 0.54 1.37 |
| Seen conflicting information across sources | **1.76** | **1.16 2.68** | **1.73** | | **1.14 2.61** | 1.36 | 0.90 2.07 | 1.34 | 0.88 2.02 |

OR: odds ratio, CI: confidence interval

logistic regressions taking depressive and anxiety symptoms (binary mental health outcomes) as dependent variables and predictors including demographic and socioeconomic factors, COVID-19-related worries (individual worries and composite worry score), general and specific daily life difficulties (individual difficulties and composite difficulty scores), support activities, levels of knowledge, sources of information, and trust in these sources, and reported confidence and satisfaction in governmental and health agencies’ responses as independent variables.

*adjusted for age, gender, change in income, and marital status.



^⁋^ adjusted for age, gender, change in income, marital status, education (university degree) and current student status.
