# Supplementary figures and images for: Mental health during the COVID-19 pandemic and first lockdown in Lebanon: Risk factors and daily life difficulties in a multiple-crises setting
Source: PLoS One. 2024 Feb 16;19(2):e0297670. doi: 10.1371/journal.pone.0297670 (PMC10871500; doi:10.1371/journal.pone.0297670)

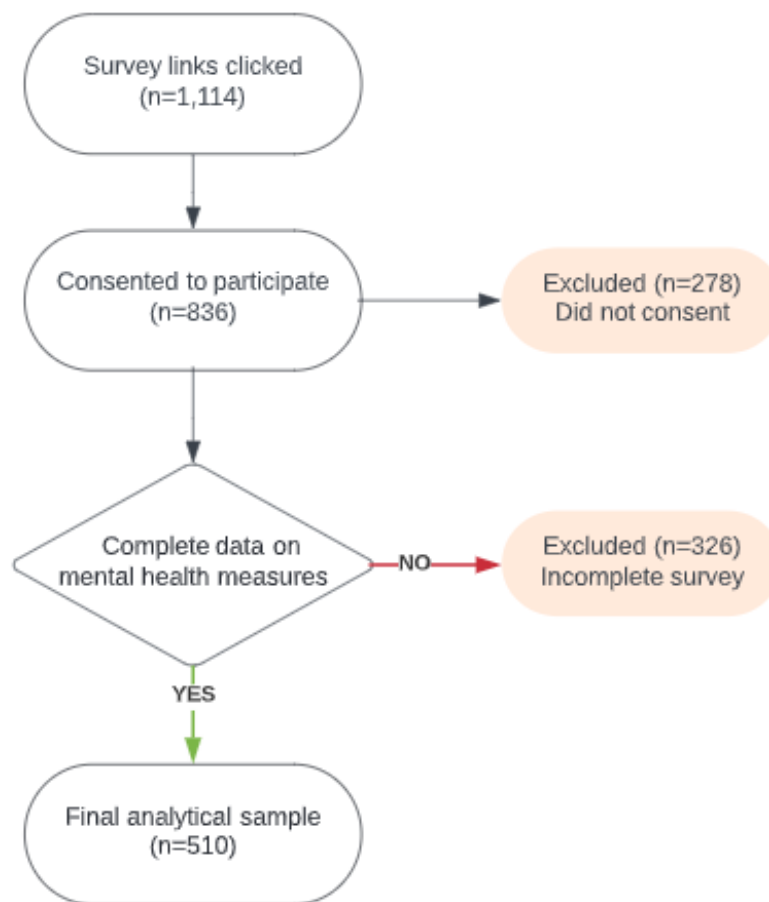

**S1 Fig.** Flow chart for the selection of participants.

Supplement: S1 Fig — (PDF) [file pone.0297670.s007.pdf]
