# Supplementary material for: Mental health during the COVID-19 pandemic and first lockdown in Lebanon: Risk factors and daily life difficulties in a multiple-crises setting
Source: PLoS One. 2024 Feb 16;19(2):e0297670. doi: 10.1371/journal.pone.0297670 (PMC10871500; doi:10.1371/journal.pone.0297670)
Supplement: S2 Fig — (PDF) [file pone.0297670.s008.pdf]

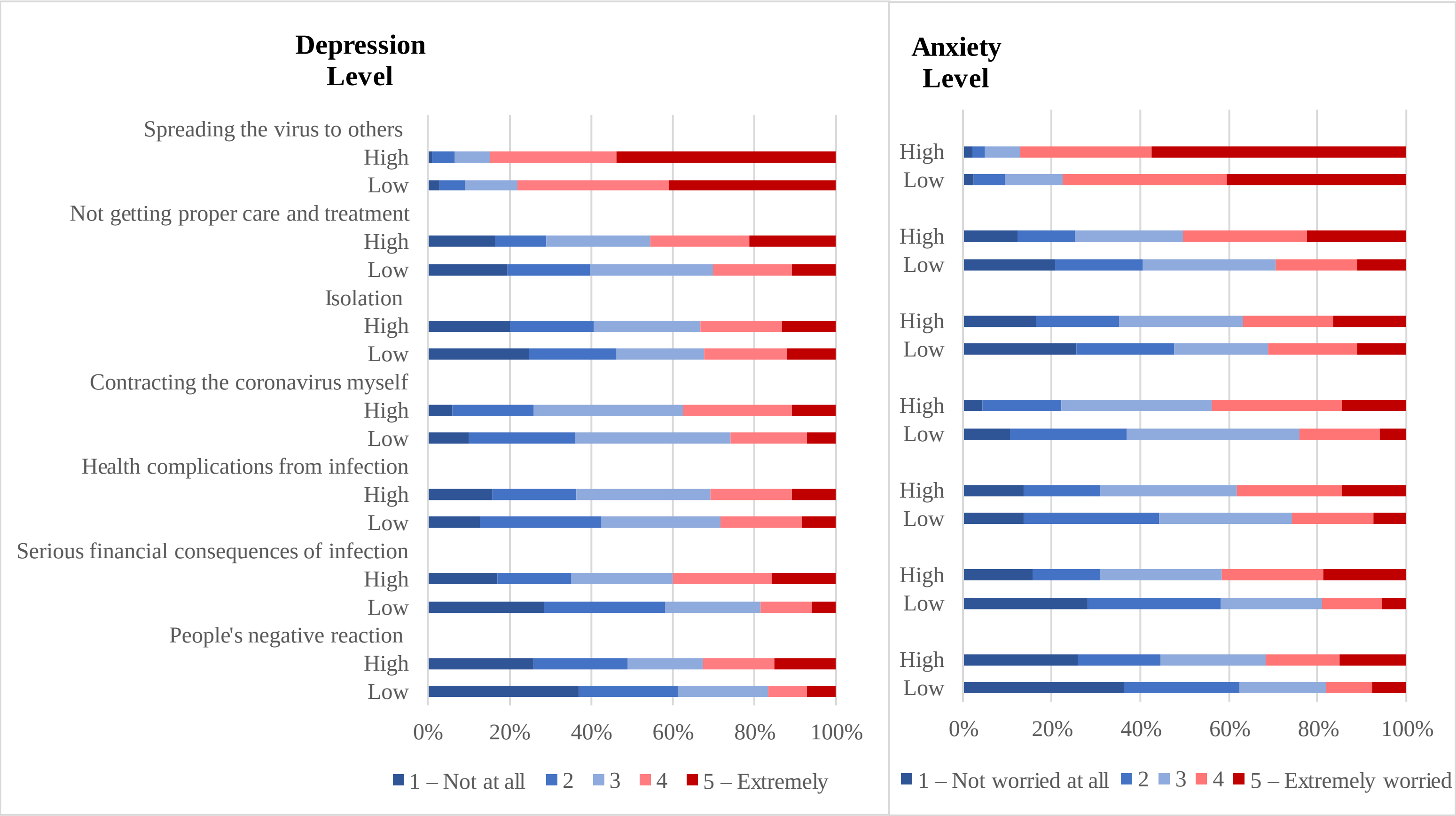

S2 Fig. Distribution of COVID-19-related worries in participants with and without elevated depression and anxiety scores.
