# Supplementary material for: Mental health during the COVID-19 pandemic and first lockdown in Lebanon: Risk factors and daily life difficulties in a multiple-crises setting
Source: PLoS One. 2024 Feb 16;19(2):e0297670. doi: 10.1371/journal.pone.0297670 (PMC10871500; doi:10.1371/journal.pone.0297670)
Supplement: S3 Fig — (PDF) [file pone.0297670.s009.pdf]

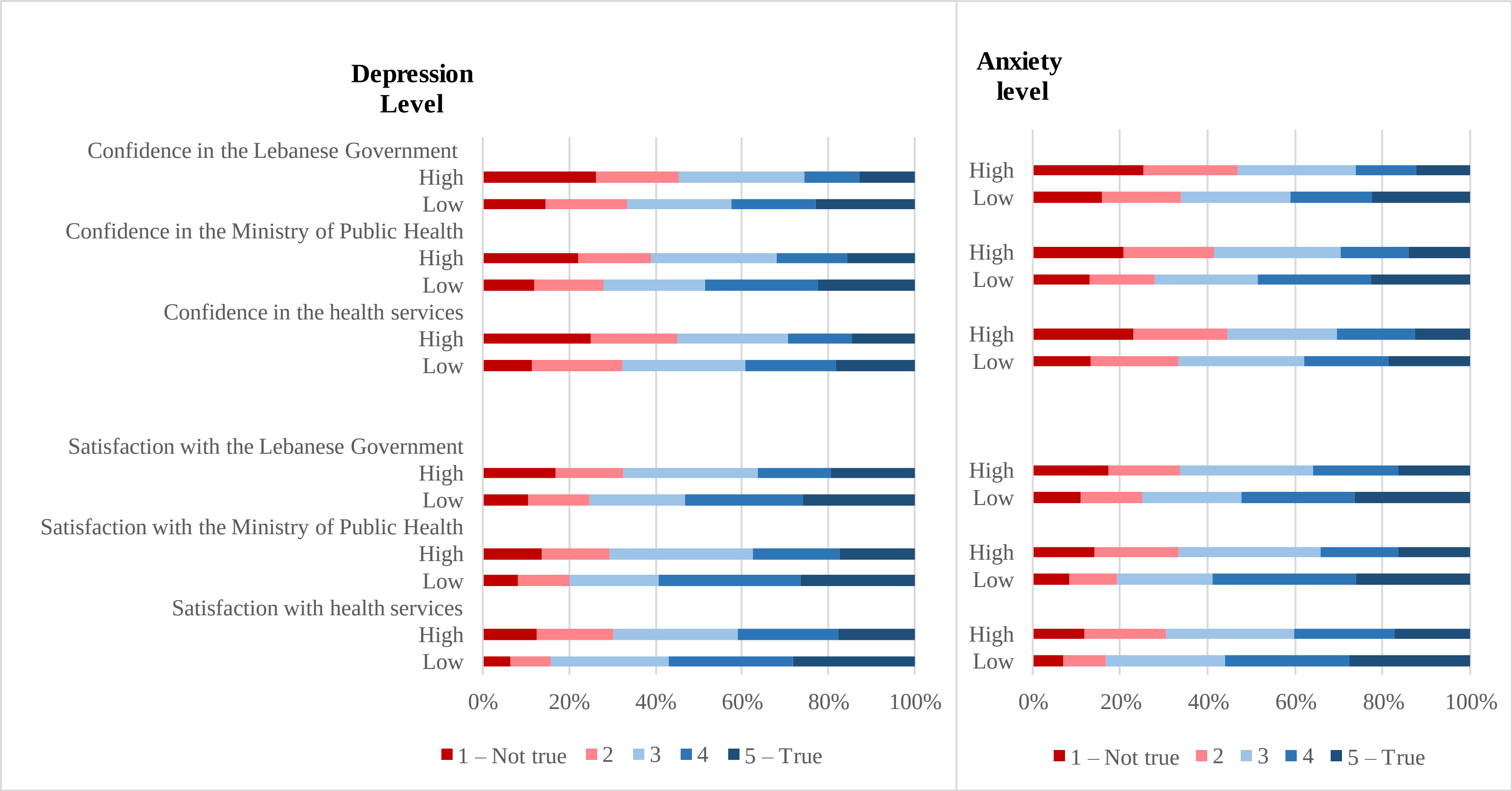

S3 Fig. Confidence and Satisfaction with the different sector's handling of the pandemic in participants with and without elevated depression and anxiety scores.
